# *JAK2* Alterations in Acute Lymphoblastic Leukemia: Molecular Insights for Superior Precision Medicine Strategies

**DOI:** 10.3389/fcell.2022.942053

**Published:** 2022-07-12

**Authors:** Charlotte EJ. Downes, Barbara J. McClure, Daniel P. McDougal, Susan L. Heatley, John B. Bruning, Daniel Thomas, David T. Yeung, Deborah L. White

**Affiliations:** ^1^ Blood Cancer Program, Precision Cancer Medicine Theme, South Australian Health and Medical Research Institute (SAHMRI), Adelaide, SA, Australia; ^2^ School of Biological Sciences, Faculty of Sciences, University of Adelaide, Adelaide, SA, Australia; ^3^ Adelaide Medical School, Faculty of Health and Medical Sciences, University of Adelaide, Adelaide, SA, Australia; ^4^ Institute for Photonics and Advanced Sensing (IPAS), University of Adelaide, Adelaide, SA, Australia; ^5^ Australian and New Zealand Children’s Oncology Group (ANZCHOG), Clayton, VIC, Australia; ^6^ Department of Haematology, Royal Adelaide Hospital and SA Pathology, Adelaide, SA, Australia

**Keywords:** leukemia, Janus kinases, kinase inhibitor, JAK2, targeted therapy, acute lymphoblastic leukemia

## Abstract

Acute lymphoblastic leukemia (ALL) is the most common pediatric cancer, arising from immature lymphocytes that show uncontrolled proliferation and arrested differentiation. Genomic alterations affecting Janus kinase 2 (*JAK2*) correlate with some of the poorest outcomes within the Philadelphia-like subtype of ALL. Given the success of kinase inhibitors in the treatment of chronic myeloid leukemia, the discovery of activating *JAK2* point mutations and *JAK2* fusion genes in ALL, was a breakthrough for potential targeted therapies. However, the molecular mechanisms by which these alterations activate JAK2 and promote downstream signaling is poorly understood. Furthermore, as clinical data regarding the limitations of approved JAK inhibitors in myeloproliferative disorders matures, there is a growing awareness of the need for alternative precision medicine approaches for specific *JAK2* lesions. This review focuses on the molecular mechanisms behind ALL-associated *JAK2* mutations and *JAK2* fusion genes, known and potential causes of JAK-inhibitor resistance, and how *JAK2* alterations could be targeted using alternative and novel rationally designed therapies to guide precision medicine approaches for these high-risk subtypes of ALL.

## Introduction

Acute lymphoblastic leukemia (ALL) is the most common pediatric malignancy, but despite cure rates now approaching 90% with refined chemotherapy regimens, relapse remains the leading cause of mortality in children (Hunger and Mullighan, 2015; Iacobucci and Mullighan, 2017; Khan et al., 2018). Furthermore, only 30–40% of adult ALL patients achieve long-term remission (Jabbour et al., 2015; Terwilliger and Abdul-Hay, 2017). Over the last decade, technological advances in genomic profiling, such as transcriptome and whole genome sequencing, have transformed risk stratification and treatment approaches for ALL patients by revealing the genomic basis of the disease (Roberts and Mullighan, 2015; Khan et al., 2018). In 2009, large-scale gene expression profiling identified a high-risk B-cell precursor ALL (B-ALL) subtype, termed Philadelphia chromosome-like ALL (Ph-like ALL), which displays a gene expression profile similar to that of Philadelphia chromosome-positive ALL (Ph+ ALL), harbors a high frequency of *IKZF1* (IKAROS family zinc finger 1) alterations, but lacks the hallmark *BCR::ABL1* (breakpoint cluster region protein/Abelson 1) fusion gene of Ph+ ALL (Mullighan et al., 2009b; Den Boer et al., 2009). Comprehensive genomic profiling studies revealed the diversity of genomic alterations that constitute the heterogeneous genomic landscape of Ph-like ALL (Tasian et al., 2017b; Pui et al., 2017; Khan et al., 2018; Iacobucci and Roberts, 2021). These genomic alterations can include translocations, cryptic rearrangements, mutations, and copy number variations, often in genes that regulate cytokine receptor and kinase signaling pathways. Genes commonly rearranged include *ABL1/2*, *CRLF2* (cytokine receptor like factor 2), *EPOR* (erythropoietin receptor) and *JAK2* (Janus kinase 2). Activating mutations or deletions are usually identified within JAK/STAT (Janus kinase/signal transducer and activator of transcription) or RAS/MAPK (RAS GTP-activating protein/mitogen-activated protein kinase) signaling pathways, although other rare kinase alterations have been reported (Roberts et al., 2012; Roberts et al., 2014a; Roberts and Mullighan, 2015; Tran and Loh, 2016).

**FIGURE 1:**
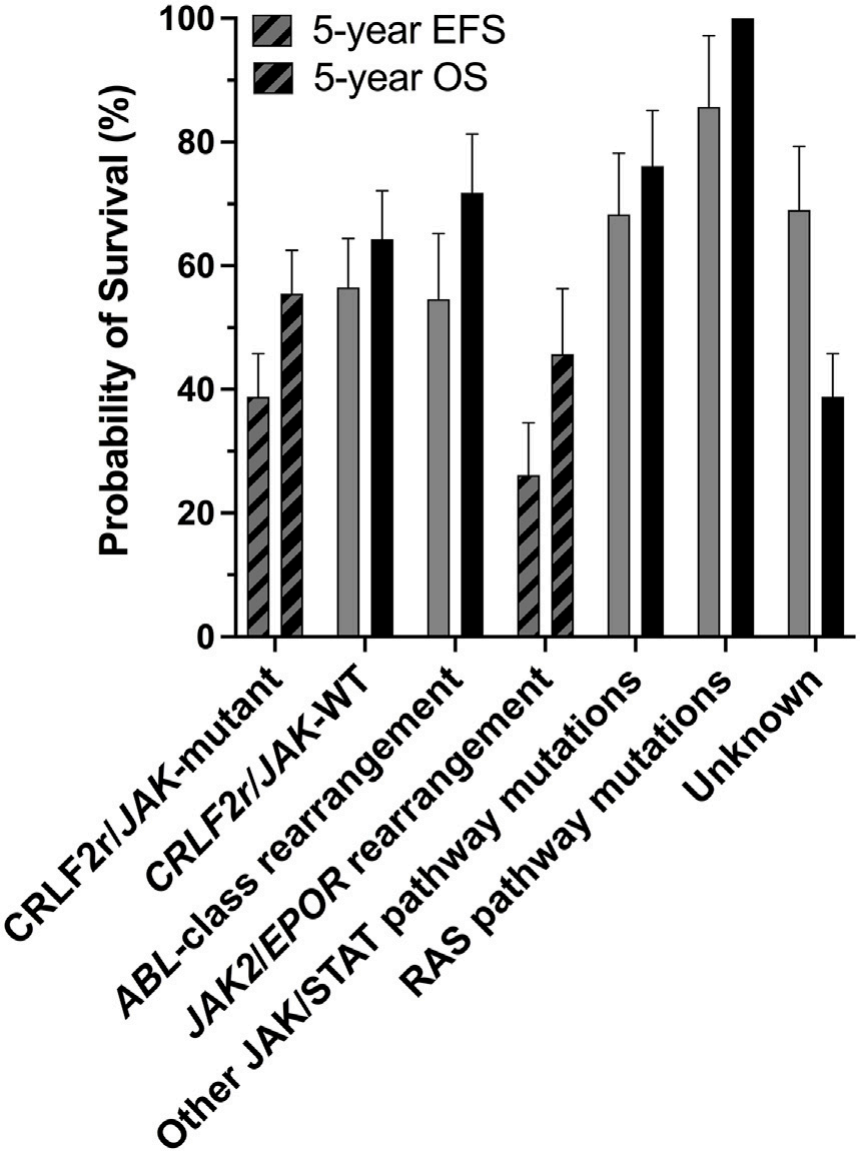
The *CRLF2*r/*JAK*-mutant and *JAK2*/*EPOR*r subtypes of Ph-like ALL are associated with poor outcomes. Outcome analyses for different genomic subtypes of Ph-like ALL for all ages combined, probabilities of 5-years event-free survival (EFS) and overall survival (OS) are shown. There are significant differences in the 5-years EFS and OS of *CRLF2*r/*JAK*-mutant and *JAK2*/*EPOR*r cases compared with other Ph-like ALL subtypes. Figure adapted from Roberts *et al.* (2014a).

*JAK2* alterations, including rearrangements and gain-of-function mutations, are associated with poor outcome within the Ph-like ALL subtype (Roberts et al., 2014a). It is unclear why *JAK2* alterations are predominantly identified within B-ALL rather than T-cell ALL (T-ALL), although there have been rare reports in T-ALL (Lacronique et al., 1997; Peeters et al., 1997; Cheng et al., 2017; Huang et al., 2020; Kaplan et al., 2021). *JAK2* chromosomal rearrangements (*JAK2*r) which result in *JAK2* fusion genes, correlate with some of the lowest survival rates within the Ph-like ALL subtype (Figure 1) (Roberts et al., 2014a; Roberts K. G. et al., 2017). Similar to oncogenic kinase drivers observed in myeloid disorders, *JAK2* alterations were identified to be driving ALL lesions, offering renewed hope for precision medicine approaches beyond high intensity combination chemotherapy. Based on the success of tyrosine kinase inhibitors (TKIs) for the treatment of chronic myeloid leukemia (CML) and Ph+ ALL, there is potential for targeted JAK2 inhibitors to improve outcomes for patients with high-risk, *JAK2*-altered ALL. The semi-selective JAK1/2 inhibitor, ruxolitinib, is currently being assessed in a number of clinical trials (NCT02723994, NCT03117751, NCT03571321, NCT02420717) for the treatment for ALL after promising efficacy was demonstrated in several pre-clinical models (Maude et al., 2012; Roberts et al., 2014a; Roberts KG. et al., 2017).

In 2011, ruxolitinib was the first JAK inhibitor approved for the treatment of myelofibrosis (MF), a myeloproliferative neoplasm (MPN) that harbors a high frequency of the recurrent activating *JAK2* p. V617F point mutation. Although ruxolitinib reduces the symptomatic burden of MF, unfortunately, it does not significantly reduce the mutant allele frequency (Deininger et al., 2015; Greenfield et al., 2018; Bewersdorf et al., 2019). Furthermore, the use of ruxolitinib as a first-line therapy for MF has revealed several clinical limitations (also apparent with a subsequently approved JAK inhibitor fedratinib), which are directly relevant to ALL and are discussed in detail below. There have been few case reports to date documenting successful responses to ruxolitinib in Ph-like ALL, with only one report in *JAK2*-mutant Ph-like ALL (Mayfield et al., 2017) and four reports in *JAK2*r Ph-like ALL (Ding et al., 2018; Chen X. et al., 2019; Chen et al., 2022; Rizzuto et al., 2022). It is also difficult to decipher the role of graft-versus-leukemia effect in the context of allogeneic transplant and the “on-target” but “off-cancer” effects of ruxolitinib on the immune system. Here, we comprehensively review the molecular biology and clinical knowledge of *JAK2* alterations in ALL. The therapeutic implications of current and future precision medicine approaches for this high-risk subtype of ALL are discussed, emphasizing the need for further lesion-specific molecular insights and a new suite of JAK-targeting approaches.

## Normal JAK2 Structure and Function

**FIGURE 2:**
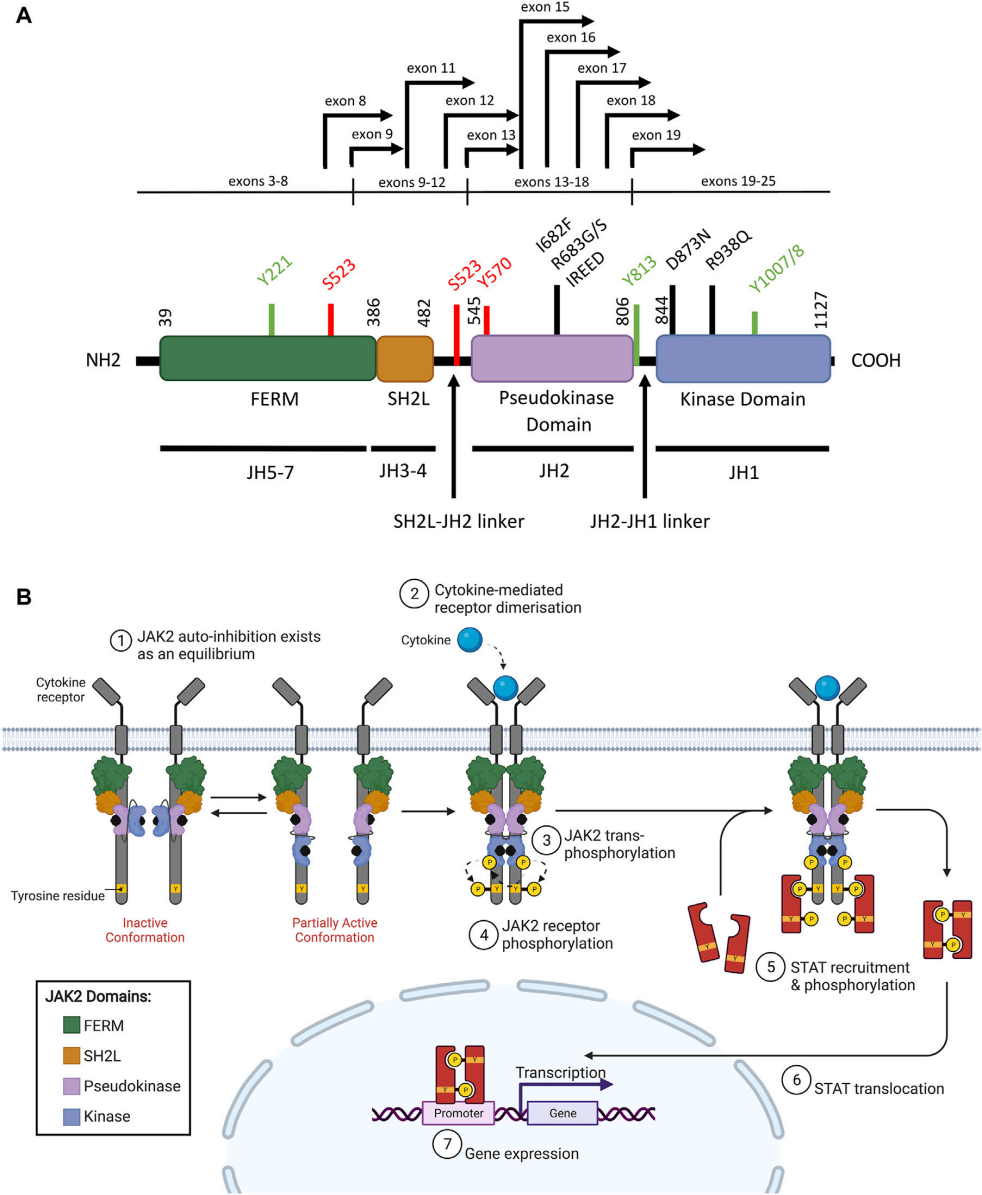
WT JAK2 structure and function **(A)** Schematic representation of the JAK2 domain structure (NCBI reference sequence: NP_004963.1) encoded by the seven JAK homology (JH) domains. The FERM (4.1 protein, ezrin, radixin, moesin), SH2 (Src homology 2)-like (SH2L), pseudokinase (JH2) and kinase (JH1) domains are represented by purple, red, light blue, and dark blue respectively. Key residues for phosphorylation for positive (green) or negative (red) regulation are shown. Mutations commonly associated with ALL (black lines) and *JAK2* fusion breakpoints (black arrows) are indicated. Adapted from Silvennoinen and Hubbard (2015b) (Silvennoinen and Hubbard, 2015a). **(B)** Schematic representation of JAK/STAT signaling pathway activation through JAK2. The JAK2 FERM and SH2L domains associate with the cytoplasmic juxtamembrane motifs of a cytokine receptor (grey) to recruit JAK2 to the cell membrane. The four domains of JAK2 are presented: FERM (green), SH2-like (orange), pseudokinase (JH2, purple), and kinase (JH1, blue). JAK2 is shown bound to ATP (black). The proposed model of JAK2 activation suggests that JAK2 exists in an equilibrium between inactive and partially active conformations. In the inactive conformation (left), the JAK2 kinase domain is inhibited by a JH2-mediated autoinhibitory interaction. In the partially active conformation (right), the JAK2 kinase domain is released from the JH2-mediated auto-inhibition and is available for some limited transphosphorylation. Cytokine (cyan) binding to their cognate receptor promotes receptor dimerisation, which facilitates JAK2 activation by transphosphorylation (arrows). JAK2 then auto-phosphorylates the cytoplasmic region of the receptor creating recruitment sites for cytoplasmic STATs (red). JAK2-mediated STAT phosphorylation facilitates STAT dimerisation. These STAT dimers are then translocated to the nucleus where they regulate gene expression by binding to promoters with STAT-binding sites. Adapted from Hubbard (2018) and “Cytokine Signaling through the JAK-STAT Pathway” (BioRender.com, 2021).

Wild-type (WT) JAK2 is a non-receptor tyrosine kinase that pre-associates with a variety of type I (containing a WSXWS motif) and type II (lacking a WSXWS motif) cytokine receptors. Upon cytokine binding, both classes of cytokine receptors activate downstream intracellular signaling pathways, predominantly the JAK/STAT signaling pathway (Babon et al., 2014; Morris et al., 2018). The JAK family kinases (comprising JAK1, JAK2, JAK3, and TYK2 (tyrosine kinase 2)) all share a common protein structure comprising seven JAK homology (JH) domains (Gnanasambandan and Sayeski, 2011; Steeghs et al., 2017). The FERM (4.1 protein, ezrin, radixin, moesin) domain and Src homology 2 (SH2)-like (SH2L) domains are N-terminally located (Figure 2A), and mediate protein-protein interactions and cytokine binding respectively (Kesarwani et al., 2015; Leroy and Constantinescu, 2017). The FERM and SH2L domains are required for JAK2 binding to specific juxtamembrane Box1 and Box2 motifs of associated cytokine receptors (Saharinen et al., 2000; Hubbard, 2018; Morris et al., 2018; Raivola et al., 2021). Phosphorylation of conserved tyrosine residues within the FERM domain can also positively or negatively regulate JAK2 activity (Gnanasambandan and Sayeski, 2011; Hammaren et al., 2019b). At the C-terminal end of JAK2 are the catalytic kinase (JH1), and pseudokinase (JH2) domains (Figure 2A) (Kesarwani et al., 2015; Leroy and Constantinescu, 2017). The kinase domain is responsible for catalyzing the phosphorylation of substrate-specific tyrosine residues (Babon et al., 2014; Morris et al., 2018). The pseudokinase domain lies directly upstream of the kinase domain, sharing conserved motifs, but exhibits minimal catalytic activity (Ungureanu et al., 2011; Lupardus et al., 2014). The pseudokinase domain allows a basal level of kinase activity to be maintained in the absence of cytokine through direct inhibition of the kinase domain (Saharinen et al., 2000; Saharinen and Silvennoinen, 2002; Hubbard, 2018). The JH2-SH2 linker region has been hypothesized to stabilize the interaction between the pseudokinase and kinase domains during this JH2-mediated auto-inhibition (Babon et al., 2014; Shan et al., 2014). Release of JH2-mediated auto-inhibition plays an important role in facilitating full JAK2 activation upon cytokine binding, as discussed below, and as such, this mechanism is often exploited by leukemogenic drivers.

### Physiological JAK2 Activation

Precise activation of cytokine receptor signaling via JAK2 is essential for the complex co-ordination of hematopoietic cell proliferation and differentiation (Vainchenker and Constantinescu, 2013; Raivola et al., 2021). In the traditional model of cytokine-induced receptor activation, high affinity cytokine binding to cognate receptor subunits results in conformational changes that facilitate and stabilize receptor dimerization and, in many cases, oligomerization of higher order protein complexes at the cell surface (Livnah et al., 1999; Vainchenker and Constantinescu, 2013). If the cytokine receptor is a homodimer then JAK2 will homodimerize, whereas heterodimeric cytokine receptors enable either JAK2 homodimerization, or heterodimerization with other JAK family members (Morris et al., 2018; Raivola et al., 2021). In contrast, several biochemical studies have reported pre-dimerization of JAK2-associated receptors, including EPOR and human growth hormone receptor (hGHR), in which cytokine binding may facilitate receptor reorientation and subsequent JAK2 activation (Livnah et al., 1999; Constantinescu et al., 2001; Gent et al., 2002; Hammaren et al., 2019b). However, the use of over-expression systems or cysteine cross-linking may have confounded such data by enriching the cell-surface density of expressed receptors (Hubbard, 2018; Wilmes et al., 2020) and thus the mechanism for pre-formed receptor activation remains speculative (Hammaren et al., 2019b). Indeed, recent single-molecule imaging by Wilmes et al. (2020) identified almost no co-trajectories of thrombopoietin receptor (TPOR) (a Type I receptor), EPOR or hGHR acting as a stable “single dimeric molecule” over time in the absence of cytokine (Wilmes et al., 2020).

In a model first proposed by Silvennoinen and Hubbard (2015a), inactive and partially active dimers of JAK2 may exist in an equilibrium at the cell membrane, where the inactive conformation is stabilized by JH2-mediated auto-inhibition (Figure 2B) (Shan et al., 2014; Silvennoinen and Hubbard, 2015a; Hubbard, 2018). In the inactive conformation, the JAK2 pseudokinase domain binds the kinase domain in a front-to-back orientation to inhibit kinase activity, a conformation stabilized by *trans*-phosphorylation of *JAK2* p. S523 and p. Y570 by the JAK2 pseudokinase domain (Saharinen et al., 2000; Saharinen and Silvennoinen, 2002; Bandaranayake et al., 2012; Shan et al., 2014; Hubbard, 2018; Hammaren et al., 2019a). In the partially active conformation, the JAK2 kinase domain is released from the JH2-mediated auto-inhibition potentially through loosening of the linker region between the SH2L and pseudokinase domains (Gnanasambandan and Sayeski, 2011; Shan et al., 2014). The partially active conformation of JAK2 is proposed to support limited *trans*-phosphorylation in the absence of cytokine to maintain a low, basal level of JAK2 activity (Shan et al., 2014; Silvennoinen and Hubbard, 2015a; Hubbard, 2018). In the traditional model, where JAK2-associated receptors do not exist as pre-formed dimers, auto-inhibition of the JAK2 kinase domain by the pseudokinase domain likely occurs in *cis* (within the same JAK2 molecule) (Hubbard, 2018). However, the disordered JAK2 JH2-JH1 linker region could be long enough to enable *trans* phosphorylation of the JAK2 kinase domain in a pre-formed receptor dimer (Hubbard, 2018). Potentially, JAK2 activation requires both cytokine-mediated receptor dimerization and release of the JH2-mediated auto-inhibitory interaction, facilitating *trans*-phosphorylation of a string of tyrosine residues located on the JAK2 activation loop, including *JAK2* p. Y1007 and p. Y1008 (Figure 2A, Figure 2B) (Feng et al., 1997; Chatti et al., 2004; Silvennoinen and Hubbard, 2015a). However, the mechanism by which these individual phosphorylation events activate JAK2 is yet to be fully elucidated (Babon et al., 2014; Hammaren et al., 2019b).

**FIGURE 3:**
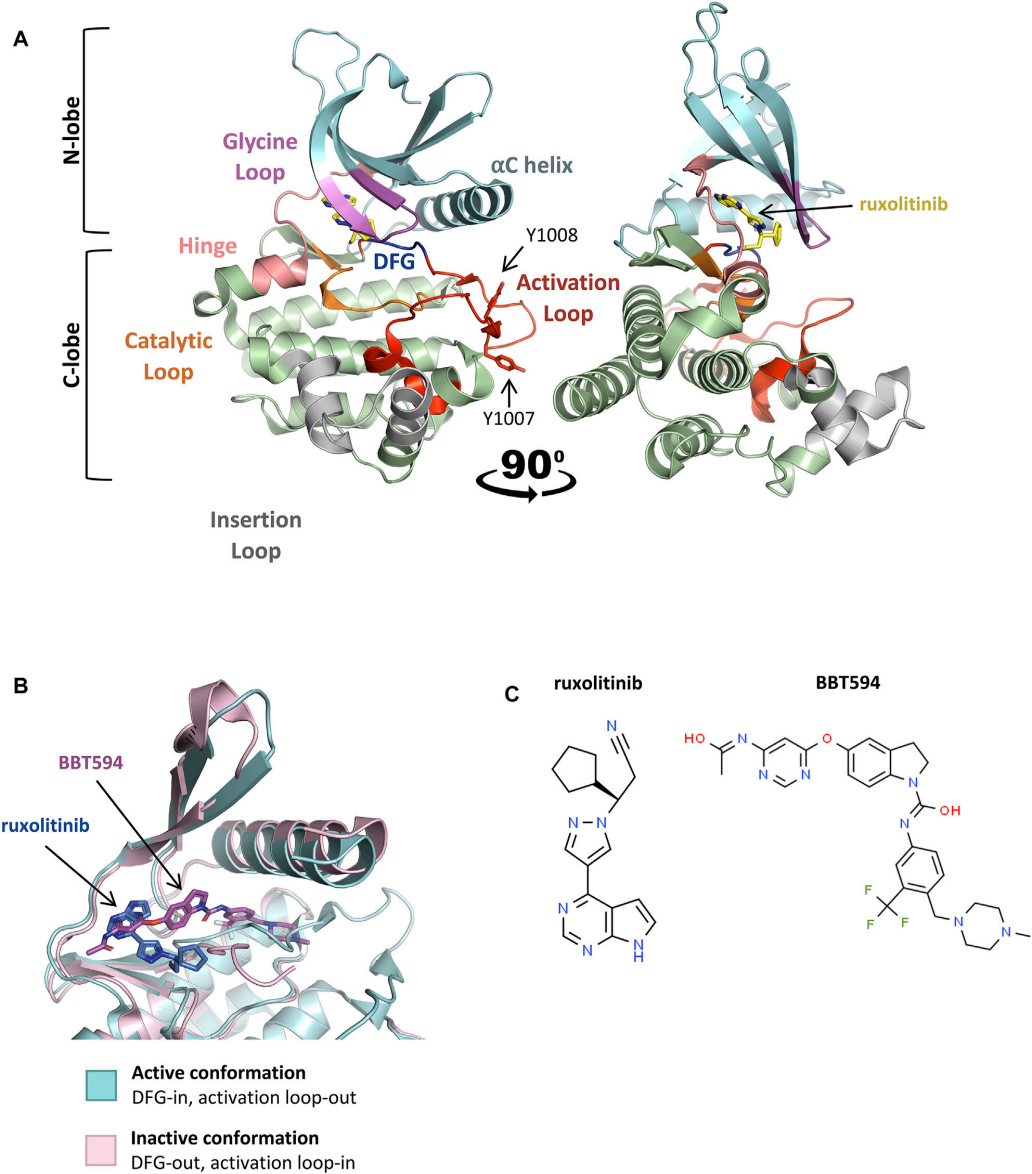
Active and inactive conformations of JAK2. **(A)** Co-crystal structure of the type-I JAK inhibitor, ruxolitinib, bound to the JAK2 kinase domain in the active conformation (PDB: 6VGL). Ruxolitinib (yellow) is presented in ball-and-stick representation with nitrogen atoms in blue. The JAK2 kinase domain is presented in ribbon representation with amino acid side chains shown for essential phosphotyrosine residues, *JAK2* p. Y1007 and p. Y1008. The N-terminal lobe (residues 840–931), shown in cyan, comprises a 5-stranded antiparallel β-sheet and one α-helix (αC). The C-terminal lobe (residues 932–1,132), shown in green, comprises 8 α-helices, 3 3/10 helices, and 3 pairs of antiparallel β-strands. The glycine loop is colored in pink, the hinge region between the 2 lobes in peach, the catalytic loop in orange, the activation loop in red, DFG-motif in blue, and the insertion loop in grey. **(B)** Superimposition of the active (blue) and inactive (pink) conformations of the JAK2 kinase domain ATP-binding site within co-crystal structures bound to JAK inhibitors. Ruxolitinib (dark blue) and the type-II JAK inhibitor, BBT594 (dark pink), are bound to the active (PDB: 6VGL) and inactive (PDB: 3UGC) conformations respectively. JAK inhibitors are presented in ball-and-stick representations with oxygen atoms in red and nitrogen atoms in blue. The JAK2 activation loop is disordered in the inactive conformation. Structures were visualized with PyMOL 2.0 (Schrödinger, LCC). **(C)** 2D chemical structures of ruxolitinib (ChemSpider, CSID: 25027389) and BBT594 (ChemSpider, CSID: 34980928)

JAK2 dimerization and *trans*-phosphorylation orientates the overall JAK2 structure to an active, or “DFG-in” conformation, characterized by the positioning of the N-lobe αC helix, and the DFG motif (residues 994–996) at the N-terminus of the activation loop (Figure 3A, Figure 3B) (Shan et al., 2014; Leroy and Constantinescu, 2017). In the active conformation, the DFG motif faces inward to enable hydrophobic interactions with the αC helix and catalytic loop (Shan et al., 2014; Leroy and Constantinescu, 2017). This rotates the αC helix towards the active site for catalysis and extends the activation loop outward to enable substrate binding (Lucet et al., 2006; Babon et al., 2014). This contrasts the inactive, or “DFG-out” conformation of JAK2, where the αC helix is rotated away from the active site and the activation loop is disordered (Figure 3B) (Silvennoinen and Hubbard, 2015b). The active conformation of JAK2 promotes ATP (adenosine triphosphate) to bind within the critical ATP-binding site, which lies between the N- and C-terminal lobes of the kinase domain (Lucet et al., 2006; Bandaranayake et al., 2012). ATP binding is stabilized by hydrogen bonds with residues located in the JAK2 hinge region and positions the terminal phosphates of ATP for phosphoryl transfer (Bandaranayake et al., 2012; Hammaren et al., 2015; Bhullar et al., 2018). A number of residues within the ATP-binding site are conserved between JAK family members, suggesting that they are essential for JAK kinase activity (Lucet et al., 2006; Bhullar et al., 2018). Following activation, JAK2 auto-phosphorylates cytoplasmic receptor tyrosine residues generating docking sites for proteins containing SH2 domains, including STATs (Figure 2B) (Levy and Darnell, 2002; Morris et al., 2018).

### The JAK/STAT Signaling Pathway

The JAK/STAT pathway is the primary signaling pathway activated by JAK2, regulating the transcription of numerous genes involved in critical pleiotropic cell processes, particularly cell proliferation, differentiation and apoptosis (Vainchenker and Constantinescu, 2013; Brachet-Botineau et al., 2020). While many JAK/STAT genes are expressed ubiquitously, mouse knockout and patient data suggest the JAK/STAT pathway is critically involved in stimulatory (rather than inhibitory) responses of immune effector cells in both innate and adaptive immunity, including mucosal cell integrity (Ye et al., 2019). JAK3 is critical for γ_c_ receptor signaling in T cells and natural killer cells, resulting in severe immunodeficiency if mutated, whereas JAK1 and TYK2 have more pleiotropic roles (Ye et al., 2019; Musella et al., 2021). JAK2 activation occurs in response to a variety of cytokines and is essential for a plethora of normal cellular functions, particularly those involved in normal hematopoiesis (Levine et al., 2007; Vainchenker and Constantinescu, 2013; Akada et al., 2014). *JAK2* knockout is embryonic lethal in mice (Neubauer et al., 1998), and is critical for signaling through homo-dimeric type-I cytokine receptors and some heterodimeric type-I receptors (Morris et al., 2018; Raivola et al., 2021). Juvenile mice with conditional *JAK2* homozygous knockout demonstrate a rapid increase in HSC apoptosis and subsequent lethality (Akada et al., 2014; Fasouli and Katsantoni, 2021; Raivola et al., 2021). STAT1 and STAT2 are critical for suppressing intracellular viral and mycobacterial infections through type-I interferon receptors; STAT3 is critical for regulating immunoglobulin E production; STAT4 and STAT6 for CD4^+^ T-helper 1 and T-helper 2 responses in adaptive immunity, respectively; while STAT5a and STAT5b are more pleiotropic in function with roles in both myeloid cell proliferation and differentiation as well as mammary gland development mediated by prolactin (Benekli et al., 2003; Awasthi et al., 2021).

Inactive STATs exist primarily in the cytoplasm as anti-parallel, inactive homo- and hetero-dimers, formed through interactions between the coiled-coil (CC) domain and the DNA-binding domain of two different STAT monomers (Neculai et al., 2005; Morris et al., 2018). The anti-parallel conformation of these inactive STAT dimers places their SH2 domains on opposing ends, accessible for binding to SH2 phosphotyrosine docking sites on cytokine receptors (Mao et al., 2005; Neculai et al., 2005). The SH2 domains of different STAT proteins determine their affinity for different cytokine receptors (Woldman et al., 2001; Ivashkiv and Hu, 2004). Receptors that activate JAK2 predominantly bind STAT5 and STAT3, which are activated by JAK2-mediated phosphorylation of a single, conserved tyrosine residue at the C-terminal end, Y705 in STAT3 (Schaefer et al., 1997), Y694 in STAT5a (Barber et al., 2001), and Y699 in STAT5b (Azam et al., 1995)). The SH2 domains of each STAT monomer then reorientate to bind this phosphorylation site in the other monomer, facilitating a conformation change to produce parallel, active dimers with exposed DNA-binding domains (Figure 2B) (Babon et al., 2014; Morris et al., 2018). These now active STAT dimers are translocated and retained in the nucleus where they act as transcription factors to regulate gene expression (Schindler and Darnell, 1995; Vainchenker and Constantinescu, 2013). The promoter regions of these genes often harbor conserved STAT-binding motifs with interferon gamma-activated site (GAS)-like core sequences (Kang et al., 2013; Brachet-Botineau et al., 2020). JAK2 also activates other signaling pathways including RAS/MAPK, phosphatidylinositol-4,5-bisphosphate 3-kinase/protein kinase B (PI3K/PKB), and mammalian target of rapamycin (mTOR) pathways (Morris et al., 2018).

Strict regulation of JAK2 activity via a variety of negative feedback loops ensures an appropriate cellular response to cytokines (Babon et al., 2014; Hammaren et al., 2019b). The suppressor of cytokine signaling (SOCS1) and SOCS3 are the key intermolecular JAK2 negative regulators that are upregulated by JAK/STAT signaling (Starr et al., 1997; Babon et al., 2014; Hammaren et al., 2019b). All SOCS family proteins contain a central SH2 domain and a short C-terminal SOCS box domain (Kershaw et al., 2013b; Morris et al., 2018). The SH2 domains of SOCS1/3 bind specific phosphotyrosine motifs to inhibit JAK1/2 and TYK2, but not JAK3 (Babon et al., 2008; Liau et al., 2018). The SOCS box domain recruits the adaptor complex, elonginBC, RING-finger-domain-only protein (RBX2) and E3 ligase scaffolds, Cullins (Babon et al., 2008; Babon et al., 2009; Zhang et al., 2015). These E3 ligase complexes catalyze the polyubiquitination and subsequent proteasomal degradation of proteins bound by the SOCS SH2 domains including JAK2 or more commonly, its associated cytokine receptors (Babon et al., 2009; Babon et al., 2014; Zhang et al., 2015). SOCS1 and SOCS3 also contain a short kinase inhibitory region (KIR) motif upstream of their SH2 domains, which can inhibit JAK2 activity by sterically hindering substrate binding (Krebs and Hilton, 2001; Kershaw et al., 2013b). The KIR is an unstructured domain that by undergoing a conformation change, can bind within the JAK2 hydrophobic substrate binding pocket with non-ATP-competitive kinetics (Krebs and Hilton, 2001; Kershaw et al., 2013a). This enables simultaneous targeting of JAK2-associated receptors for degradation and direct JAK2 inhibition (Kershaw et al., 2013a; Kershaw et al., 2013b; Liau et al., 2018).

Another SH2-domain containing protein, the lymphocyte adaptor protein (LNK or SH2B3), also negatively regulates JAK2 activity (Babon et al., 2014; Morris et al., 2018). LNK inhibits JAK2 activity by directing binding regulatory JAK2 phosphotyrosine residues including *JAK2* p. Y813, which lies within the JH1-JH2 linker region (Maslah et al., 2017). LNK may also inhibit signaling activation through JAK2 by competitively binding critical cytoplasmic phosphotyrosine residues on cytokine receptors (Maslah et al., 2017). Furthermore, JAK/STAT signaling can be negatively regulated by protein tyrosine phosphatases (PTPs), which dephosphorylate critical tyrosine residues within JAK2, STATs or JAK2-associated cytokine receptors (Bohmer and Friedrich, 2014). Cytoplasmic phosphatases that regulate JAK2 include protein tyrosine phosphatase non-receptor type 1 (PTPN1 or PTP1B), type 6 (PTPN6 or SHP1) and type 11 (PTPN11 or SHP2) (Babon et al., 2014). PTPN6 is primarily expressed in hematopoietic cells and inhibits JAK2 by binding and dephosphorylating *JAK2* p. Y429 within the JAK2 SH2-like domain (Klingmüller et al., 1995; Bohmer and Friedrich, 2014). In contrast, PTPN11 is ubiquitously expressed and can positively or negatively regulate JAK/STAT signaling in different contexts (Hammaren et al., 2019b). JAK/STAT signaling can also be regulated through receptor phosphatases such as protein tyrosine phosphatase receptor type C (PTPRC or CD45) and type T (PTPRT) (Babon et al., 2014; Morris et al., 2018). PTPRT can dephosphorylate *STAT3* p. Y705 (Zhang et al., 2007), whereas CD45 is highly expressed in hematopoietic cells and has been demonstrated to dephosphorylate all JAK family proteins in murine cells (Irie-Sasaki et al., 2001), and JAK1 and JAK3 in human cells (Yamada et al., 2002; Bohmer and Friedrich, 2014). The suite of JAK2 regulators highlights the critical role of strict JAK2 control for appropriate responses to cytokine stimulation in normal cells.

## *JAK2* Mutations in Ph-like ALL

Appropriate regulation of JAK/STAT signaling plays a critical role in the development and functional activation of crucial hematopoietic cells, including hematopoietic stem cells (HSCs) (Wang and Bunting, 2013; Fasouli and Katsantoni, 2021; Raivola et al., 2021). The importance of JAK2 in hematological malignancies became apparent in 2005 after four research groups identified a single missense mutation within the pseudokinase domain of JAK2; *JAK2* p. V617F, as the primary driving alteration underlying most MPNs (Baxter et al., 2005; James et al., 2005; Kralovics et al., 2005; Levine et al., 2005; Silvennoinen and Hubbard, 2015a; Hubbard, 2018). Following identification of *JAK2* p. V617F in 2005 (Baxter et al., 2005; James et al., 2005; Kralovics et al., 2005; Levine et al., 2005), an array of other *JAK2* mutations have been identified in MPNs, myeloma, lymphoma, and chronic and acute leukaemias of either myeloid or lymphoid lineage (Lee et al., 2006; Krämer et al., 2007; Furqan et al., 2013; Vainchenker and Constantinescu, 2013; Fasouli and Katsantoni, 2021; Raivola et al., 2021). Gain-of-function mutations in *JAK2* have been identified in the high-risk Ph-like ALL subtype, occurring exclusively with rearrangements of *CRLF2* (*CRLF2*r), which lead to *CRLF2* overexpression (Roberts et al., 2012; Roberts et al., 2014a; Boer et al., 2017; Tasian et al., 2017b; Pui et al., 2017; Reshmi et al., 2017; Steeghs et al., 2017). Approximately 50% of Ph-like ALL patients harbor *CRLF2*r, and roughly half of these patients also harbor activating point mutations in *JAK1* or *JAK2* (Table 1) (Mullighan et al., 2009a; Mullighan et al., 2009c; Russell et al., 2009; Pui et al., 2017; Reshmi et al., 2017). *JAK* alterations also occur in approximately 20% of Down-Syndrome ALL (DS-ALL) patients, with *CRLF2*r identified in approximately 60% of DS-ALL patients (Bercovich et al., 2008; Mullighan et al., 2009c; Hertzberg et al., 2010; Schwartzman et al., 2017; Page et al., 2018; Harvey and Tasian, 2020).

**TABLE 1:** Frequency of *JAK2* mutations and *JAK2* rearrangements within Ph-like ALL. Prevalence of *CRLF2*r/*JAK* mutant and *JAK2*r subtypes of Ph-like ALL compared with Ph-like ALL cases without *CRLF2* overexpression.

Clinical trial	Age (years)	Total (N)	Non-*CRLF2*	*CRLF2*r *JAK* WT	*CRLF2*r *JAK* mutant	*JAK2*r	References
**AALL0232**	1–18	31	8	10	12	1	Loh *et al.* (2013)
Multiple trials*							Roberts *et al.*, 2014a
	1–15 (SR)	33	25	5	3	0	
	1–15 (HR)	108	57	19	26	6	
	16–20	77	27	14	32	4	
	21–39	46	15	17	7	7	
**GMALL**	15–65	16	5	5	6	N/A**	Herold *et al.* (2017)
**University Pennsylvania**							Tasian *et al.*, 2017a
	18–39	7	2	3	2	0	
	40–88	11	2	4	5	0	
**Multiple trials**							
	21–39	96	41	35	14	6	Roberts *et al.*, 2017a
	40–59	62	27	24	5	6	
	60–86	36	13	15	6	2	
**St. Jude Total XV**	1–18	40	29	5	6	0	Roberts *et al.*, 2014b

*Multiple trials include cohorts from St. Jude’s Children’s Research Hospital, the Children’s Oncology Group (COG), the Eastern Cooperative Oncology Group (ECOG), M.D., Anderson Cancer Center (MDACC), and the Alliance for Clinical Trials in Oncology (Cancer and Leukemia Group B, CALGB).

**Data not available.

### *JAK2* Exon 14 Mutations and the Molecular Activation Mechanisms of *JAK2* p. V617F

**FIGURE 4:**
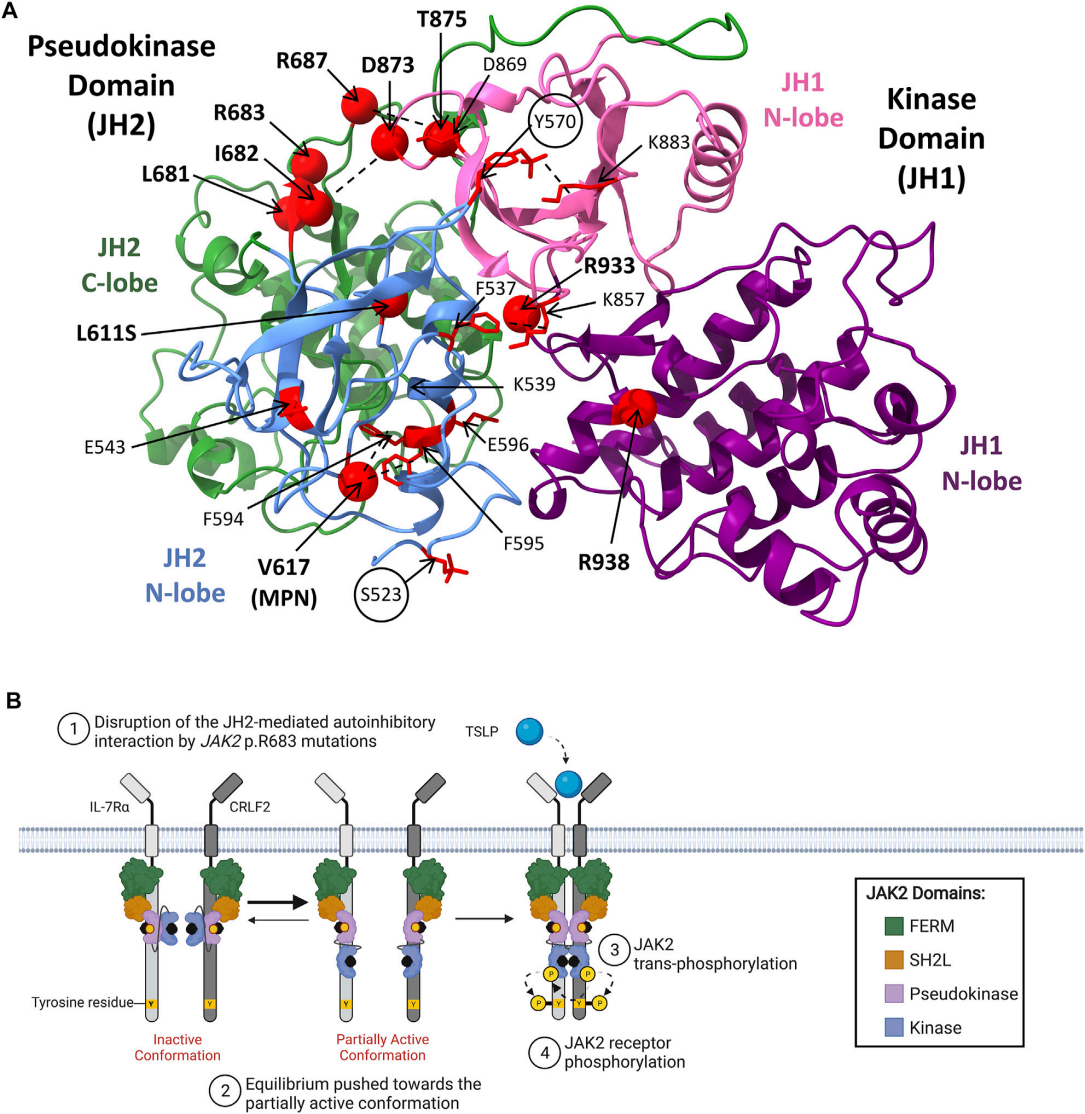
JAK2 mutations in hematological malignancies. **(A)** Model of JAK2 JH2-JH1 interface showing the positions of known activating JAK2 mutations. The JAK2 JH2-JH1 model was generated by Shan *et al.* (2014) using molecular dynamics simulations and annotated using ChimeraX-1.2.5 (University of California). The JH2 (pseudokinase domain) N-terminal (residues 536–629) and C-terminal (residues 630–839) lobe are colored in light blue and green respectively. The JH1 (kinase domain) N-terminal (residues 840–931) and C-terminal (932–1,132) lobes are colored in pink and purple respectively. Residues that when mutated are known to be activating are shown as red spheres (α carbon). Two critical inhibitory phosphorylation sites, pS523 and pY570, are encircled. Other key residues predicted to be involved in activating JAK2 mechanisms are colored in red and presented with amino acid side chains shown. Proposed interactions are represented by dashed lines. Figure adapted from Shan *et al.* (2014), Hammarén *et al.* (2019a), Leroy *et al.* (2016) and Lupardus *et al.* (2014). **(B)** Schematic representation of JAK/STAT signaling pathway activation through mutant-JAK2. CRLF2 (dark grey) heterodimerizes with IL-7Rα (light grey) to form the cytokine receptor for TSLP. The JAK2 FERM and SH2-like domains associate with the cytoplasmic juxtamembrane motifs of the receptor to recruit JAK2 to the cell membrane. The four domains of JAK2 are presented: FERM (green), SH2-like (orange), pseudokinase (JH2, purple), and kinase (JH1, blue). JAK2 is shown bound to ATP (black). The proposed model of mutant-JAK2 activation suggests that mutations such as *JAK2* p. R683G (represented by a yellow sphere) disrupt the JH2-mediated autoinhibitory interaction with the kinase domain. This shifts the equilibrium of JAK2 from the inactive, auto-inhibited state towards the partially active state, supporting mutant-JAK2 dimerisation. Although mutant-JAK2 alone remains dependent on cytokine binding, additional mechanisms such as receptor overexpression may promote malignant transformation. Adapted from “Cytokine Signaling through the JAK-STAT Pathway” (BioRender.com, 2021).

Most *JAK2* mutations associated with hematological malignancies encode missense mutations that localize within *JAK2* exon 12 of SH2L-JH2 linker region, or within *JAK2* exons 14 or 16 of the pseudokinase domain, highlighting these regions as oncogenic hot-spots for mutation (Figure 4A) (Mullighan et al., 2009c; Gnanasambandan and Sayeski, 2011; Silvennoinen and Hubbard, 2015b). Mutations within *JAK2* exon 14 associate primarily with MPNs, where *JAK2* p. V617F occurs in >95% of patients with polycythemia vera (PV), and ∼60% of patients with essential thrombocythemia (ET) or primary myelofibrosis (PMF) (Baxter et al., 2005; James et al., 2005; Kralovics et al., 2005; Levine et al., 2005; Vainchenker and Constantinescu, 2013; Silvennoinen and Hubbard, 2015a). Interestingly, the *JAK2* p. V617F mutation has not been identified in ALL and only a single *JAK2* exon 14 mutation, *JAK2* p. L611S, has been reported in an ALL setting (Kratz et al., 2006; Funakoshi-Tago et al., 2009; Gnanasambandan and Sayeski, 2011; Jain et al., 2017; Konoplev et al., 2017). This suggests that *JAK2* exon 14 mutations associate primarily with myeloid lineage diseases. Different JAK2 mutants have demonstrated varying affinities to lineage-specific cytokine receptors, which may explain phenotypic differences induced by different *JAK2* mutations and their association with either myeloid or lymphoid lineage diseases (Yao et al., 2017).

Mutagenesis studies have demonstrated that *JAK2* p. V617F confers cytokine-independent signaling activation (Baxter et al., 2005; James et al., 2005; Kralovics et al., 2005; Senkevitch and Durum, 2017). Activating *JAK2* mutations, including *JAK2* p. V617F, were initially predicted to confer cytokine-independent signaling through disruption of the JH2-mediated autoinhibitory interaction, facilitating mutant-JAK2 dimerization (Gnanasambandan and Sayeski, 2011; Ungureanu et al., 2011; Hubbard, 2018; Glassman et al., 2022). In addition, a recent report using single-molecule microscopy demonstrated that the *JAK2* p. V617F mutation confers cytokine-independent dimerization of receptor subunits (50% of the maximum level for TPOR, 25% for EPOR and 10% for hGHR), with a stable time-dependent dimer formation similar to cytokine binding (Wilmes et al., 2020). However, introduction of *JAK2* p. V617F into JAK2 JH2-JH1 protein fragments revealed that the *JAK2* p. V617F mutation only resulted in a 3-fold increase in JAK2 catalytic activity, while deletion of the pseudokinase domain produced a 20-fold increase (Sanz et al., 2011). This suggested that disruption of the JH2-mediated autoinhibitory interaction alone is not sufficient to constitutively activate signaling through JAK2 (Hammaren et al., 2019a; Hammaren et al., 2019b). Leroy *et al.* (2016) and Glassman *et al.* (2022) have proposed two molecular mechanisms for JAK2 constitutive activation by *JAK2* p. V617F (Leroy et al., 2016; Glassman *et al.*). The first mechanism destabilizes the JH2-JH1 autoinhibitory interaction through the formation of a π stacking interaction between *JAK2* p. V617F and *JAK2* p. F595, which stabilizes the JAK2 pseudokinase domain αC helix in a straightened conformation (Bandaranayake et al., 2012; Leroy et al., 2016; Hubbard, 2018). The second mechanism involves the formation of a positive regulatory interaction that favors dimerisation of active JAK2 monomers (Leroy et al., 2016; Glassman *et al.*). The combination of this positive regulatory interaction, in addition to the disruption of the JH2-mediated autoinhibition, may explain the high driving activity of the *JAK2* p. V617F mutation (Hammaren et al., 2019a; Hammaren et al., 2019b).

### *JAK2*Disruption of JH2-Mediated Autoinhibition and the role of *CRLF2* Overexpression

In contrast to *JAK2* p. V617F, the molecular mechanisms by which *JAK2* mutations identified ALL disrupt JH2-mediated autoinhibition and facilitate JAK2 dimerization are yet to be fully elucidated (Hammaren et al., 2019a). *JAK2* mutations reported in patients with ALL and DS-ALL are shown in Table 2. The transformative ability of all ALL-associated *JAK2* mutations are dependent on cytokine receptor association, suggesting that these mutations are dependent on JAK2 dimerization (Lu et al., 2008; Wernig et al., 2008; Yao et al., 2017; Hammaren et al., 2019b). The majority of ALL-associated *JAK2* mutations lie within *JAK2* exon 16 (Table 2), where the most frequent mutations are *JAK2* p. R683G/S (Mullighan et al., 2009a; Harvey et al., 2010; Pui et al., 2017; Kim et al., 2018). *JAK2* exon 16 mutations all localize to the ATP-binding site of the JAK2 pseudokinase domain between the N- and C-terminal lobes (Figure 4A) (Gnanasambandan and Sayeski, 2011; Ungureanu et al., 2011; Bandaranayake et al., 2012). ATP binding to the JAK2 pseudokinase domain is essential for JAK2 activation as mutations within this region are known to suppress JAK2 kinase activity (Hammaren et al., 2015; Hammaren et al., 2019a). *JAK2* p. R683 maps to the JAK2 pseudokinase domain β7-β8 loop and forms an ionic interaction with *JAK2* p. D873 within the JAK2 kinase domain β2-β3 loop (Figure 4B) (Hammaren et al., 2019a; Hammaren et al., 2019b). Mutations of *JAK2* p. R683 (Table 2) are predicted to disrupt this ionic interaction within the JH2-JH1 interface and hinder the JH2-mediated autoinhibitory interaction (Lupardus et al., 2014; Shan et al., 2014; Hammaren et al., 2019a; Hammaren et al., 2019b). Similarly, mutations of *JAK2* p. L681 and p. I682 are predicted to alter the positioning of *JAK2* p. R683, thereby disrupting JH2-mediated autoinhibition by affecting its interaction with *JAK2* p. D873 (Li et al., 2015).

**TABLE 2:** All reported *JAK2* mutations in patients with ALL. The majority of mutations reported in ALL and DS-ALL localize to *JAK2* exon 14 or 16, encoding the JAK2 pseudokinase domain (JH2). Some mutations have also been reported to localize to *JAK2* exon 20 or 21, encoding the JAK2 kinase domain (JH1). Amino acids (aa.) encoded by each *JAK2* exon are shown.

Domain location	Exon location (Aa encoded)	Mutation	ALL/DS-ALL	References
**Pseudokinase Domain (JH2)**	**Exon 14 (593–622)**	L611S	ALL	Kratz et al. (2006), Funakoshi-Tago et al. (2009), Gnanasambandan and Sayeski (2011), Jain et al. (2017), Konoplev et al. (2017)
	**Exon 16 (665–710)**	L681-I682ins	DS-ALL	Bercovich *et al.* (2008)
		TPYEGMPGH		
		I682F	ALL	Mullighan et al. (2009c), Suryani et al. (2015), Jain et al. (2017), Konoplev et al. (2017)
		I682del insMPAP	DS-ALL	Bercovich *et al.* (2008)
		R683G	ALL, DS-ALL	Bercovich et al. (2008), Mullighan et al. (2009c), Gaikwad et al., 2009, Kearney et al. (2009), Suryani et al. (2015), Jain et al. (2017), Konoplev et al. (2017)
		R683S	ALL, DS-ALL	Bercovich et al. (2008), Mullighan et al. (2009c), Gaikwad et al. (2009), Kearney et al. (2009), Jain et al. (2017), Konoplev et al. (2017)
		R683K	DS-ALL	Bercovich *et al.* (2008)
		R683T	DS-ALL	Gaikwad *et al.* (2009)
		QGinsR683	ALL	Mullighan *et al.*, 2009c
		2GinsR683	ALL	Malinge *et al.* (2007)
		GPinsI683	ALL	Suryani *et al.* (2015)
		I682-D686 del	DS-ALL	Malinge *et al.* (2007)
		R687Q	ALL	Mullighan et al. (2009c), Suryani et al. (2015), Jain et al. (2017)
**Kinase**	**Exon 20 (858–920)**	D873N	ALL	Mullighan et al. (2009c), Suryani et al. (2015), Jain et al. (2017)
		T875N	ALL	Jain *et al.* (2017)
**Domain (JH1)**	**Exon 21 (921–962)**	P933R	ALL	(Mullighan et al., 2009c; Suryani et al., 2015; Kim et al., 2018)
		R938Q	ALL	(Marty et al., 2014; Sadras et al., 2017)

While *JAK2* mutations associated with ALL are predicted to disrupt JH2-mediated autoinhibition, these mutations alone are not sufficient to constitutively activate JAK2 (Hammaren et al., 2019a; Hammaren et al., 2019b). Instead, release of this autoinhibitory interaction may support the partially active conformation of JAK2, potentially exposing an interface to facilitate JAK2 dimerization (Figure 4A) (Hubbard, 2018). The high association of *JAK1/2* mutations with *CRLF2*r (Mullighan et al., 2009a; Mullighan et al., 2009c; Russell et al., 2009; Pui et al., 2017; Reshmi et al., 2017) in ALL suggests that these events functionally cooperate to drive lymphoid transformation (Russell et al., 2009; Tasian and Loh, 2011; Kim et al., 2018). *CRLF2* overexpression has been demonstrated to increase the proliferation of primary lymphoid progenitors (Russell et al., 2009). However, similar to *JAK1/2* mutations, *CRLF2* overexpression alone is not sufficient to transform cytokine-dependent cells (Russell et al., 2009; Roll and Reuther, 2010). Several groups discovered that murine pro-B cells expressing human *CRLF2* can only drive cytokine-independent proliferation when co-expressed with ALL-associated *JAK2* mutations (Mullighan et al., 2009a; Mullighan et al., 2009c; Hertzberg et al., 2010; Roll and Reuther, 2010; Yoda et al., 2010). As further support, a more recent study using transgenic mice demonstrated that while expression of *CRLF2* alone in B-lineage hematopoietic cells did not induce B-ALL development, *CRLF2* transgenic mice transplanted with fetal liver cells expressing *JAK2* p. R683G or *JAK2* p. P933R-mutant *JAK2* succumbed to ALL disease within 10–20 days post-transplantation (Kim et al., 2018). These studies suggest that *CRLF2*r and *JAK2* mutations cooperate to drive leukaemogenesis (Russell et al., 2009; Tasian and Loh, 2011; Kim et al., 2018), a fact which could be exploited for therapeutic advantage in Ph-like ALL. CRLF2 heterodimerizes with interleukin 7 receptor alpha chain (IL-7Rα) to form the thymic stromal lymphopoietin receptor (TSLPR) (Tasian and Loh, 2011; Bugarin et al., 2015; Page et al., 2018), and *CRLF2*r highly correlate with increased TSLPR surface expression (Bugarin et al., 2015; Konoplev et al., 2017; Pastorczak et al., 2018). Potentially, the combination of increased TSLPR expression and an increased ratio of JAK2 in the partially active conformation resulting from *JAK2* mutations, cooperate to drive a leukaemic transformation.

There have also been some rare activating *JAK2* mutations identified in ALL that localize to the JAK2 kinase domain (Mullighan et al., 2009c; Marty et al., 2014; Sadras et al., 2017; Hammaren et al., 2019b). These include *JAK2* p. D873N, p. T875N, p. P933R, and p. R938Q (Mullighan et al., 2009c; Marty et al., 2014; Suryani et al., 2015; Jain et al., 2017; Sadras et al., 2017). *JAK2* p. D873N and p. T875N that localize to *JAK2* exon 20, encoding part of the JAK2 ATP-binding site that lies in the proximity of the glycine loop (Lucet et al., 2006). *JAK2* p. D873N is expected to activate JAK2 through loss of its ionic interaction with *JAK2* p. R683, weakening the JH2-JH1 autoinhibitory interaction to facilitate JAK2 dimerization (Chen C. et al., 2019; Hammaren et al., 2019a). Likewise, *JAK2* p. T875N is proposed to weaken the JH2-JH1 autoinhibitory interaction via an allosteric mechanism involving the disruption of a hydrogen bond with *JAK2* p. D873 (Dusa et al., 2010; Gnanasambandan and Sayeski, 2011; Chen C. et al., 2019). *JAK2* exon 21 mutations, *JAK2* p. P933R and p. R938Q, are also expected to disrupt the JH2-JH1 autoinhibitory interaction but these mutations map to the conserved JAK2 hinge region of the ATP-binding site (Lucet et al., 2006; Marty et al., 2014). The mechanism of *JAK2* p. P933R activation is poorly understood, however, *JAK2* p.938Q has been proposed to disrupt JH2-mediated autoinhibition through loss of an ionic interaction between *JAK2* p. R867 and *JAK2* p. D869 (Marty et al., 2014). Overall, all *JAK2* mutations reported in ALL are predicted to weaken JH2-mediated autoinhibition, similar to *JAK2* p. V617F, likely increasing the probability of receptor dimerization. However, the lack of an additional second molecular mechanism driven by *JAK2* exon 16 mutations, unlike *JAK2* p. V617F, may explain why ALL-associated *JAK2* mutations require *CRLF2* overexpression to cooperatively drive malignant transformation and subsequent leukemogenesis.

## *JAK2* Rearrangements in Ph-like ALL

**FIGURE 5:**
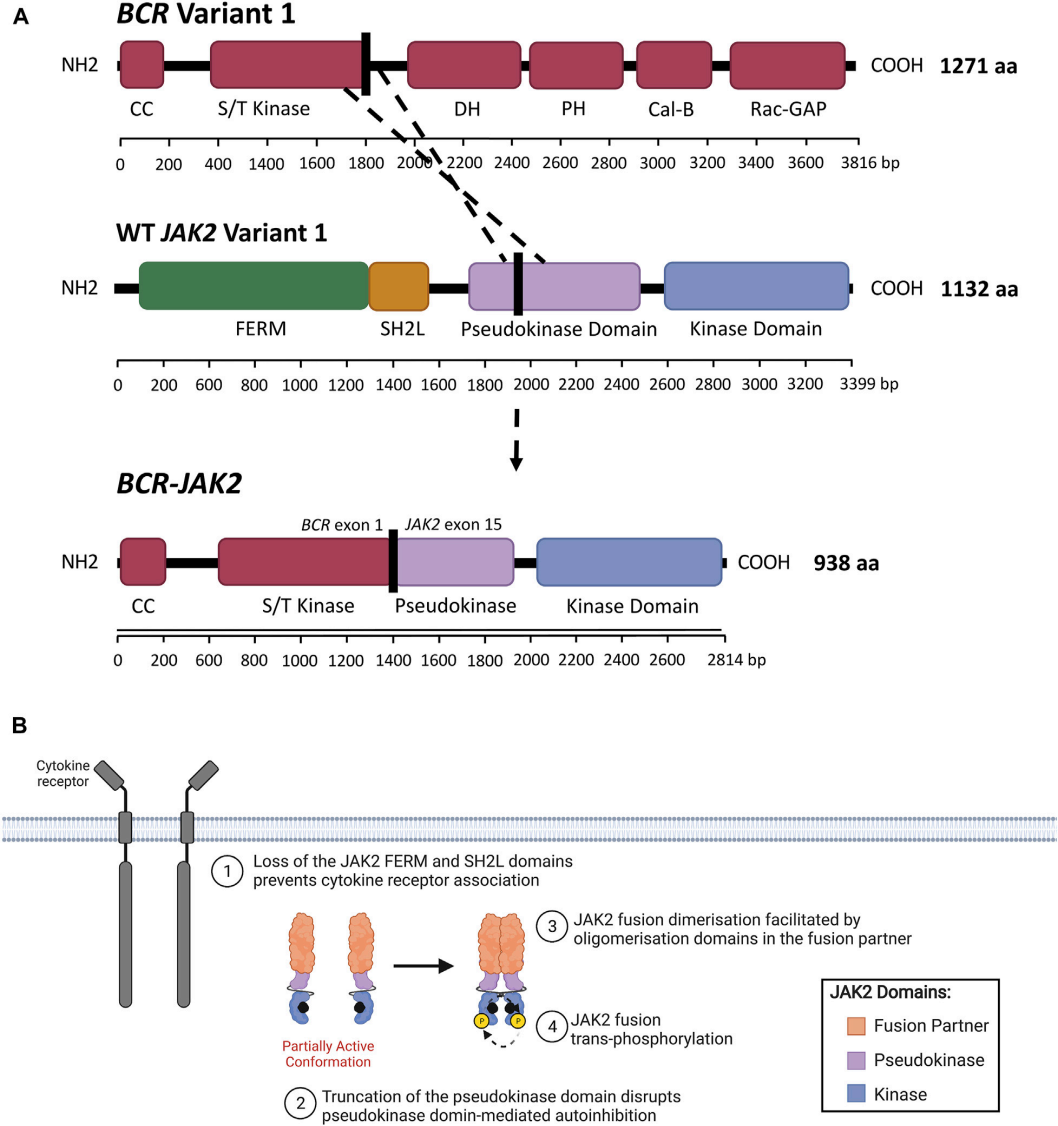
JAK2 fusion proteins in ALL. **(A)** Schematic representation of a genomic rearrangement between *JAK2* exon 15 and *BCR* exon 1 that produces the *BCR-JAK2* fusion gene. BCR isoform 1 (encoded by *BCR* variant 1) contains the following domains: BCR coiled-coil (CC), serine/threonine kinase (S/T kinase), DH (Dbl homology), PH (pleckstrin homology), Cal-B (calcium-dependent lipid-binding) and Rac-GAP (Rac GTPase-activating protein) domains. The BCR DH and PH domains form the Rho-GEF domain (Rho guanine nucleotide exchange factor). *JAK2* isoform A (encoded by *JAK2* variant 1) contains FERM (4.1 protein, ezrin, radixin, moesin), SH2-like (SH2L, Src homology 2), pseudokinase (JH2) and kinase (JH1) domains. The BCR-JAK2 fusion protein retains the BCR CC and S/T kinase domains, three exons of the JAK2 pseudokinase domain and the full-length JAK2 kinase domain. BCR-JAK2 is predicted to homodimerise via its retained BCR CC motif. Domains encoded by the *BCR*, *JAK2* and *BCR-JAK2* transcripts were annotated using InterPro (EMBL-EBI, 2021) (Jones et al., 2014; Blum et al., 2021) and Maru amd Witte (1991). **(B)** Schematic representation of JAK/STAT signaling pathway activation through JAK2 fusions. All JAK2 fusions comprise of an N-terminal fusion partner (orange) and the full-length JAK2 kinase domain (JH1, blue). The full-length or truncated JAK2 pseudokinase domain (JH2, purple) may also be present or absent in different JAK2 fusions. The absence of the JAK2 FERM and SH2-like domains prevent JAK2 fusions from associating with the cytoplasmic juxtamembrane motifs of cytokine receptors (dark grey). JAK2 fusions are shown bound to ATP (black). The proposed model of JAK2 fusion activation suggests that oligomerization domains within the fusion partner may facilitate JAK2 fusion dimerisation and subsequent *trans*-phosphorylation, promoting malignant transformation. Adapted from “Cytokine Signaling through the JAK-STAT Pathway” (BioRender.com, 2021).

In addition to *JAK2* mutations, *JAK2* rearrangements have been associated with various myeloid and lymphoid hematological malignancies (Furqan et al., 2013; Vainchenker and Constantinescu, 2013; Levavi et al., 2019; Raivola et al., 2021). The *ETV6::JAK2* (ETS variant transcription factor 6/*JAK2*) fusion (initially known as *TEL::JAK2*) was the first *JAK2*r identified by cytogenics in both ALL and CML patients 1997 and was the first *JAK2* alteration demonstrated to induce constitutive activation of JAK2 (Lacronique et al., 1997; Peeters et al., 1997; Raivola et al., 2021). The JAK2 fusion proteins encoded by these *JAK2*r comprise the N-terminus of a fusion partner and the C-terminus of JAK2 (Figure 4B) (Ho et al., 2010; Babon et al., 2014; Boer and den Boer, 2017). For example, the rearrangement between *BCR* and *JAK2* produces the *BCR::JAK2* fusion gene (Figure 5A). All reported *JAK2* fusion genes retain *JAK2* exons 19-25 encoding the kinase domain (Table 3), however the influence of the 5’ fusion partner gene is not well characterized. A diverse range of *JAK2* fusion partner genes have been reported across different lymphoid and myeloid malignancies (Levavi et al., 2019). There have been 94 reported cases of *JAK2*r in ALL (Table 3), in comparison, only four cases of *JAK2*r have been reported in MPNs, including *BCR::JAK2*, *PCM1::JAK2* (pericentriolar material 1/*JAK2*), *RPN1::JAK2* (ribophorin 1/*JAK2*) and *PEX14::JAK2* (peroxisomal biogenesis factor 14/*JAK2*) (Murati et al., 2005; Mark et al., 2006; Elnaggar et al., 2012; Lundberg et al., 2014; He et al., 2016; Levavi et al., 2019). Albeit in very low numbers, *JAK2*r have also been identified in solid tumors including breast cancer (Quesada et al., 2021) and small lung cancer (Iwakawa et al., 2013; Levavi et al., 2019), but these particular *JAK2*r have not been reported in any hematological malignancies.

**TABLE 3:** Reported *JAK2* fusion gene partners in patients with *JAK2*r ALL. For more details and corresponding references see Supplementary Table S1. The JAK2 pseudokinase domain is encoded by *JAK2* exons 13-18.

Fusion partner	N	Number of *JAK2*r ALL patients			M:F ratio	Exon break within *JAK2*
		Child	AYA	Adult		
***ATF7IP***	3		1	2	2:1	8, 16, & 17
***BCR***	12^a^	5	1	2	7:0	15, 17, & 19
***EBF1***	1		1		NA	17
***ERC1***	1^a^	NA	NA	NA	NA	NA
***ETV6***	9^a^	3	3	1	7:1	17, & 19
***GOLGA4***	1			1	1:0	12
***GOLGA5***	1	1			1:0	19
***HMBOX1***	1	NA	NA	NA	NA	NA
***MPRIP***	1	NA	NA	NA	1:0	NA
***NPHP3***	1^a^	NA	NA	NA	NA	NA
***OFD1***	2	2			2:0	13
***PAX5***	27^a^	11	4	3	3:5	18, & 19
***PCM1***	3^a^	1		1	2:1	9
***PPFIBP1***	1		1		1:0	19
***RFX3***	1	NA	NA	NA	0:1	NA
***RNPC3***	2^a^		1		0:1	13
***ROCK1***	1^a^	NA	NA	NA	NA	NA
***SMU1***	1			1	0:1	13
***SNX29***	2^a^	NA	NA	NA	NA	NA
***SPAG9***	1	1			1:0	19
***SSBP2***	6^a^	1	2	1	1:1	11, 17, & 18
***STRBP***	1		1		0:1	19
***STRN3***	2	2			0:2	17
***TBL1XR1***	1	1			1:0	14
***TERF2***	3^a^	1	1		1:1	19
***TPM3***	1		1		1:0	17
***TPR***	1		1		1:0	17
***USP25***	1	NA	NA	NA	1:0	NA
***ZBTB20***	2		2		0:2	19
***ZBTB46***	2^a^			1	0:1	19
***ZEB2***	1	NA	NA	NA	1:0	NA
***ZNF274***	1	NA	NA	NA	0:1	NA

N, total number of reported ALL, cases harboring *JAK2* fusion genes with the specified fusion partner; *JAK2*r ALL, *JAK2*-rearranged acute lymphoblastic leukemia; Child, aged <15 years; AYA, adolescent or young adult, aged 16–39 years; Adult, aged 40–86 years; NA, data not available.

a Age/sex of some patients not specified.

*JAK2*r in B-ALL are identified exclusively within the Ph-like subtype, occurring in approximately 5% of pediatric Ph-like ALL cases (<15 years) with the highest frequency in young adult patients (16–39 years) (∼14%) (Table 1) (Roberts et al., 2012; Roberts et al., 2014a; Imamura et al., 2016; Roberts K. G. et al., 2017; Boer et al., 2017; Tasian et al., 2017b; Jain et al., 2017; Reshmi et al., 2017). In MPNs, *JAK2*r are associated with a more aggressive phenotype than fusions involving other kinase genes such as *PDGFRA* (platelet-derived growth factor receptor A), and long-term remission can often only be achieved after allogenic stem cell transplantation (Allo-SCT) (Schwaab et al., 2015; Schwaab et al., 2020). Similarly, *JAK2*r in ALL are associated with the poorest outcomes compared with other Ph-like ALL subtypes (Roberts K. G. et al., 2017; Jain et al., 2017; Iacobucci and Roberts, 2021). All reported *JAK2* fusion genes retain *JAK2* exons 19-15 encoding the kinase domain (Table 3) and the chimeric JAK2 fusion proteins encoded by these *JAK2* fusion genes have demonstrated constitutive JAK2 kinase activation (Cuesta-Domínguez et al., 2012; Roberts et al., 2012; Roberts et al., 2014a; Schinnerl et al., 2015; Boer and den Boer, 2017; Steeghs et al., 2017). In contrast to *JAK2* mutations, expression of *JAK2*r in primary murine pre-B cells results in cytokine-independent proliferation, suggesting that *JAK2* fusion genes alone are driving genomic lesions in *JAK2*r ALL (Cuesta-Domínguez et al., 2012; Roberts et al., 2014a; Schinnerl et al., 2015). Over 30 different *JAK2* fusion partner genes have been identified in Ph-like ALL to date, the most common of which is *PAX5* (Paired box 5) (28.7%) (Table 3, Supplementary Table S1) (Roberts et al., 2012; Roberts et al., 2014a; Yano et al., 2015; Imamura et al., 2016; Roberts K. G. et al., 2017; Boer et al., 2017; Reshmi et al., 2017; Li et al., 2018; Schwab and Harrison, 2018; Gu et al., 2019; Tang et al., 2019). Other commonly identified *JAK2* fusion partners in ALL are *BCR::JAK2* (12.8%), *ETV6::JAK2* (9.6%), *SSBP2* (single stranded DNA binding protein 2/*JAK2*) (6.4%) and *ATF7IP* (activating transcription factor 7 interacting protein) (3.2%) (Table 3, Supplementary Table S1) (Roberts et al., 2014a; Roberts K. G. et al., 2017).

Similar to other Ph-like ALL subtypes, *JAK2*r often co-occur with deletions in genes involved in B-cell development including *IKZF1* (IKAROS family zinc finger 1) (Mullighan et al., 2008; Mullighan et al., 2009b). The most common *IKZF1* alteration associated with Ph-like (and *JAK2*r) ALL is a deletion of *IKZF1* exons 3-6, encoding the dominant negative IK6 isoform of IKAROS, which lacks the N-terminal DNA binding domain (Roberts et al., 2014a; Tran et al., 2018; Shiraz et al., 2020). IKAROS IK6 is unable to bind DNA to regulate the expression of genes required for B-cell differentiation, implying that *JAK2*r and *IKZF1* deletions both drive deregulation of B-cell maturation and promote development of B-ALL (Mullighan et al., 2009b; Harvey et al., 2010; Pui et al., 2017). *IKZF1* alterations are also associated with inferior event-free survival in Ph-like ALL patients (Mullighan et al., 2009b; Van der Veer et al., 2013; Roberts et al., 2014a). A number of other genomic alterations also co-occur with *JAK2* fusion genes and often involve B-cell pathways, including deletions of *PAX5*, *BTG1* (BTG anti-proliferation factor 1), and *CDKN2A/B* (cyclin-dependent kinase inhibitor 2A/B) (Roberts et al., 2012; Roberts et al., 2014a; Boer et al., 2015; Kawamura et al., 2015; Roberts K. G. et al., 2017). Deletions of *RAG1/2* (recombination-activating gene 1 and 2), *VPREB* (V-set pre-B cell surrogate light chain 1), *EBF1* (EBF transcription factor 1), *RUNX1* (RUNX family transcription factor 1), *BTLA* (B and T lymphocyte associated), *CD200* (CD200 molecule) and *ETV6* have also been reported to co-occur with *JAK2* fusion genes, as well as mutations within *IKZF1*, *KRAS* (KRAS proto-oncogene), *SETD1* (SET domain-containing 1A), and *PTPN11* (Roberts et al., 2012; Roberts et al., 2014a; Boer et al., 2015; Roberts K. G. et al., 2017). Although prognostic significance of some of these alterations have been demonstrated in Ph-like ALL (Tran and Loh, 2016; Roberts K. G. et al., 2017; Pui et al., 2017; Roberts et al., 2018; Tran et al., 2018; Zhang et al., 2019), the influence of these additional alterations on *JAK2*r patient survival rates is not well elucidated.

### Cytokine-Independent Oligomerization

In contrast to *JAK2* mutations, the molecular mechanism by which *JAK2* fusion genes lead to constitutive JAK2 activation remains largely unknown. The *JAK2* regions encoding the full-length JAK2 FERM domain are absent in all reported *JAK2* fusion genes (Table 3, Figure 2A). The presence of the JAK2 FERM domain has been demonstrated to be critical for JAK2 localization to the plasma membrane (Zhao et al., 2010) and JAK/STAT signaling activation (Eder-Azanza et al., 2017). The absence of FERM and SH2-like domains in *JAK2*r likely prevents binding of JAK2 fusions to membrane-associated cytokine receptors, implying these fusion products can promote signaling in the absence of cytokine. Considering the critical role of cytokine-mediated receptor dimerization in WT JAK2 activation (Silvennoinen and Hubbard, 2015a), activation of JAK2 fusions, unlike mutant-JAK2, likely occurs via a mechanism that does not require receptor association. The normal function and tissue specificity of *JAK2* fusion partner genes is diverse and their typical expression, or lack of expression, with B-cells is varied (Table 3, Supplementary Table S2). However, the majority of these JAK2 fusion partners have the ability to oligomerize (Supplementary Table S2), suggesting that JAK2 fusion activation occurs through direct homodimerization. The proposed model suggests that JAK2 fusions oligomerize via the presence of oligomerization domains within the N-terminal fusion partner (Medves and Demoulin, 2012). The most common of these oligomerization domains are CC motifs, present in 44% of JAK2 fusion partners reported in ALL, including BCR:JAK2 (Figure 5A) (Cuesta-Domínguez et al., 2012; The UniProt Consortium, 2019). These oligomerization domains may facilitate JAK2 fusion *trans*-phosphorylation (Figure 5B), however, the quaternary structure of different JAK2 fusions has not yet been elucidated and there are limited published studies investigating the functional impact of JAK2 fusion partner oligomerization domains (Medves and Demoulin, 2012).

Disruption of the BCR CC motif within the BCR:ABL1 fusion has been shown to abrogate the transformative ability of BCR:ABL1 (Beissert et al., 2008; Mian et al., 2009), suggesting that the BCR CC motif is essential for BCR:ABL oligomerization and subsequent constitutive activation. The therapeutic potential of CC mimetics is now being investigated but may be amenable to CC-containing *JAK2*r (Dixon et al., 2012; Bruno and Lim, 2015; Woessner et al., 2015; Peiris et al., 2020). The helix-loop-helix (HLH) domain, also known as the sterile alpha motif (SAM) or pointed (PNT) domain, is another oligomerization domain that can facilitate self-association (Medves and Demoulin, 2012; Hock and Shimamura, 2017). Deletion of the ETV6 HLH domain has been shown to abrogate the transforming kinase activity of ETV6:LYN (ETV6/tyrosine-protein kinase Lyn) (Takeda et al., 2011) and ETV6:JAK2 (Lacronique et al., 1997) fusion proteins. This suggests that the ETV6 HLH domain may enable constitutive activation of ETV6:JAK2 and EBF1:JAK2 fusions by facilitating JAK2 fusion homodimerization (Medves and Demoulin, 2012; Hock and Shimamura, 2017). Other domains within JAK2 fusion partners that may facilitate JAK2 fusion oligomerization include BR-C, ttk and bab (BTB) domains, scan motifs and LIS1 homology (LisH) domains, but there are likely more oligomerization domains to be identified (Supplementary Table S2) (Poitras et al., 2008; Tijchon et al., 2013). BTB domains are present in ZBTB20 (Zinc finger and BTB domain-containing 20) and ZBTB46, scan motifs in ZNF274 (Zinc finger protein 274), and LisH domains in SSBP2 (single stranded DNA binding protein 2) (Poitras et al., 2008; Tijchon et al., 2013) (Supplementary Table S2).

JAK2 fusions may also be *trans*-phosphorylated through indirect oligomerization such as via recruitment to larger protein complexes such as centrioles, spliceosomes, nuclear pore complexes (NPCs), or telomere nucleoprotein complexes (Medves and Demoulin, 2012). For example, OFD1:JAK2 ((OFD1 centriole and centriolar satellite protein/JAK2) and PCM1:JAK2 (pericentriolar material 1/JAK2) may be activated by indirect oligomerization at centriolar satellites, as both OFD1 and PCM1 are components of centrioles (Supplementary Table S2) (Medves and Demoulin, 2012; Lee and Stearns, 2013). To support this, a kinase fusion containing the centrosome protein, FGFR1 oncogene partner (FOP), was demonstrated to localize to centriolar satellites where tyrosine phosphorylation was increased (Lee and Stearns, 2013). FOP shares homology with OFD1 and co-localizes with PCM1 (Lee and Stearns, 2013). In addition, some domains and regulatory sites retained within the JAK2 fusion partner could mediate interactions that facilitate JAK2 fusion activation or contribute to leukemogenesis. For example, tyrosine residues within the fusion partner could be phosphorylated and influence intracellular signaling by enabling recruitment of proteins containing SH2 domains (Medves and Demoulin, 2012). However, the significance of these potential interactions to overall cell transformation and disease phenotype is debated (Medves and Demoulin, 2012). Further research is required to understand whether these potential interactions are retained or whether higher order protein complexes can form.

### Alternate Mechanisms of JAK2 Fusion Activation

Constitutive activation of the majority of JAK2 fusions likely occurs through a cytokine-independent oligomerization mechanism. However, unlike all other reported JAK2 fusions, PAX5:JAK2 does not harbor an oligomerization domain or self-associate, yet still constitutively activates JAK/STAT signaling similar to other *JAK2*r (Schinnerl et al., 2015; Sakamoto et al., 2017; Jurado et al., 2022). This suggests that PAX5:JAK2 may be activated via a mechanism distinct from cytokine-independent oligomerization (Schinnerl et al., 2015). PAX5:JAK2 is the only JAK2 fusion protein that has been shown to localize within the nucleus due the presence of a nuclear localization signal (NLS) within the PAX5 fusion partner (Schinnerl et al., 2015). Potentially, PAX5:JAK2 may constitutively activate JAK/STAT signaling by phosphorylation of nuclear STATs (Schinnerl et al., 2015). PAX5:JAK2 also retains the ability of PAX5 to act as a transcription factor, binding and activating PAX5 target loci through its paired domain (Schinnerl et al., 2015; Jurado et al., 2022). Similarly, the majority of JAK2 fusion partners are transcription factors containing DNA-binding domains including CC, HLH, zinc finger C2H2 type, or leucine zippers. Two other JAK2 fusion proteins, ATF7IP:JAK2 and TERF2:JAK2 (Telomeric repeat binding factor 2/JAK2), also contain NLSs but their localization has not been investigated to date, nor has their ability to bind DNA. In addition, many of these DNA-binding JAK2 fusion partners can act as tumor-suppressors, and their DNA-binding domains may also function as oligomerization domains (Medves and Demoulin, 2012). Therefore, oligomerization between JAK2 fusions and their endogenous JAK2 fusion partner may contribute to leukemogenesis by impairing the tumor-suppressive function of the WT JAK2 fusion partner (Medves and Demoulin, 2012). For example, HLH-mediated oligomerization between WT ETV6 and ETV6:JAK2 may reduce the availability of the endogenous ETV6 HLH motif, which normally maintains long-term transcriptional repression of genes by interaction with Polycomb group complexes (De Braekeleer et al., 2012).

Interestingly, a study by Fortschegger *et al.* (2014) demonstrated that PAX5:JAK2 phosphorylation occurs independently of DNA-binding or *trans*-phosphorylation by another kinase (Schinnerl et al., 2015). Fortschegger *et al.* (2014) hypothesized that the absence of the JAK2 pseudokinase domain within PAX5:JAK2 may enable constitutive activation of PAX5:JAK2 by preventing JH2-mediated pseudokinase domain auto-inhibition (Schinnerl et al., 2015). Consistent with this hypothesis, loss of the JH2-JH1 autoinhibitory interaction by either deletion of JAK2 JH2 or the destabilizing *JAK2* p. F739R mutation has been shown to increase basal JAK2 kinase activity (Saharinen et al., 2000; Saharinen and Silvennoinen, 2002; Hammaren et al., 2015). Therefore, that truncation or deletion of this domain in *JAK2* fusion genes may contribute to constitutive activation of JAK2 fusions such as PAX5:JAK2. The *JAK2* pseudokinase domain (encoded by *JAK2* exons 13–18) is either absent or truncated in most *JAK2* fusion genes (Table 3). Only four JAK2 fusions contain full-length pseudokinase domains, including GOLGA5:JAK2 (Golgin A5/JAK2) (Ding et al., 2018), OFD1:JAK2 (Yano et al., 2015; Imamura et al., 2016), RNPC3:JAK2 (RNA binding region containing 3/JAK2) (Chen X. et al., 2019; Chen et al., 2021), SMU1:JAK2 (SMU DNA Replication Regulator and Spliceosomal Factor/JAK2) (Roberts K. G. et al., 2017) (Table 3) and it is currently unknown whether these fusions display less JAK2 kinase activity in comparison to JAK2 fusions that harbor truncated or deleted pseudokinase domains. The functional effects of different truncations of the JAK2 pseudokinase domain is also unknown.

In addition, one publication reported that *JAK2* was highly expressed in pediatric *JAK2*r B-ALL patients in comparison to non-Ph-like B-ALL patients (Steeghs et al., 2017). *JAK2* is expressed at a low level in normal B-cells, in comparison to some *JAK2* fusion partner genes that are highly expressed in normal B-cells (Supplementary Table S2). This high-level expression may result from the *JAK2*r being placed under the control of the *JAK2* fusion partner’s promoter. However, no other reports specify whether *JAK2*r are highly expressed in patients and the importance *JAK2*r transcript expression levels are currently unknown. Although overexpression may be suggested to contribute the leukaemic potential of *JAK2*r, overexpression of WT *JAK2* alone is not transforming *in vitro* (Yoda et al., 2010), suggesting that *JAK2*r transcript expression levels are of marginal importance. Overall, the impact of the fusion partner within *JAK2* fusion genes is largely unknown but cytokine-independent oligomerization is predicted to be the driving mechanism behind JAK2 fusion constitutive activity. There are also several other potential mechanisms by which *JAK2* rearrangements may contribute to upregulated downstream signaling including loss of JH2-mediated autoinhibition and upregulation of gene expression. Further research assessing the biological phenotypes of different *JAK2* fusion partner genes and different breakpoints within *JAK2* is required and may potentially reveal novel regulation mechanisms.

## JAK2 as a Target for Precision Medicine in ALL

ALL patients harboring *JAK2* alterations are currently treated with multi-agent chemotherapy and corticosteroids (Terwilliger and Abdul-Hay, 2017). Allo-SCT following high-dose chemotherapy improves survival in selected patients. (Terwilliger and Abdul-Hay, 2017). However, these intense regimens result in a number of acute and chronic side effects and are accompanied by an increased risk of treatment-related mortality (Senkevitch and Durum, 2017). Intensive chemotherapy regimens are often poorly tolerated in adults and the elderly due to toxicity and an increased occurrence of co-morbidities, a contributor to poor outcomes in these age groups (Terwilliger and Abdul-Hay, 2017). Immunotherapies, such as blinatumomab and chimeric antigen receptor (CAR) T-cell immunotherapy, have proven their effectiveness as salvage therapy in B-ALL (Inaba and Pui, 2019; Zhao et al., 2019). They are now being incorporated into frontline therapy for high risk disease, and may enable the dosage and duration of chemotherapy to be reduced to alleviate toxicity (Inaba and Pui, 2019; Zhao et al., 2019). Underscoring the importance of JAK2 in the pathogenesis of ALL, Roberts *et al.* (2014a) reported 5-years event-free survival (EFS) rates of 38.8% for *CRLF2*r/*JAK*-mutant ALL patients and 26.1% for ALL patients harboring a rearrangement of either *JAK2* or *EPOR* (Figure 1) (Roberts et al., 2014a), subsequently reported as 23.5% in a later study (Roberts K. G. et al., 2017). In both studies, these 5-years EFS were significantly inferior to non-Ph-like ALL subtypes (Roberts et al., 2014a; Roberts K. G. et al., 2017). The poor outcomes associated with *JAK2* alterations in ALL highlights the urgent need for more effective and less toxic treatment strategies for these high-risk patients (Roberts and Mullighan, 2015). Targeting of JAK2 with small molecule inhibitors in combination with chemotherapy may be one such therapeutic approach, given the remarkable success of TKIs for the treatment of CML (Ali, 2016).

### TKIs as a Paradigm for Targeted Therapy

Direct inhibition of BCR:ABL1 using TKIs has served as a paradigm for the application of targeted therapies (Ali, 2016; Mughal et al., 2016). The first TKI identified to successfully inhibit BCR:ABL1 kinase activity was STI571, now known as imatinib (Druker et al., 1996; Apperley, 2015). This first-generation TKI is classified as a type-II inhibitor, as it inhibits BCR:ABL1 kinase activity by competitively binding the inactive conformation of ABL1 within the ATP-binding site (Druker and Lydon, 2000; Schindler et al., 2000; Rossari et al., 2018). CML patients who achieve a deep molecular response on imatinib therapy for 2 or more years can now expect a normal life expectancy (Gambacorti-Passerini et al., 2011), and 10-years overall survival rates have improved from less than 20% prior to 1982, to now around 83% (Druker et al., 2006; Mughal et al., 2016; Hochhaus et al., 2017). Identification of imatinib resistance, often acquired through the emergence of point mutations within the BCR:ABL1 kinase domain, has since driven the development of second- and third-generation TKIs (Zabriskie et al., 2014; Patel et al., 2017; Pottier et al., 2020; Shoukier et al., 2021). There are now six TKIs that are FDA-approved for the treatment of CML including imatinib, nilotinib, dasatinib, bosutinib, ponatinib and asciminib (Hughes et al., 2019; Shoukier et al., 2021). Incorporation of imatinib into treatment approaches for Ph+ ALL has also drastically improved EFS rates, from 27% to 72% (Senkevitch and Durum, 2017), suggesting that similar approaches may also be successful for the treatment of *JAK2*-altered ALL. The success of TKIs as a precision medicine approach for targeting BCR:ABL1 in CML and Ph+ ALL launched a new era of discovery into targeted cancer therapies (Sawyers, 2003; Rossari et al., 2018). In particular, the development of small molecule inhibitors of other constitutively active kinases were pursued to potentially treat a variety of other diseases and malignancies (Sawyers, 2003; Zhang et al., 2009; Cohen et al., 2021).

### JAK2 as a Therapeutic Target

The identification of the *JAK2* p. V617F mutation underlying the majority of MPNs positioned JAK2 as an attractive molecular target for small molecule screening and development (Constantinescu, 2009; Kumar et al., 2009). Targeted JAK2 inhibitors entered clinical development just 6 years following the first report of *JAK2* p. V617F (Levine et al., 2007; Pardanani, 2007). In 2011, the semi-selective JAK1/2 inhibitor, ruxolitinib (Figure 3C), was the first JAK2 inhibitor to be FDA-approved for the treatment of MF and hydroxyurea resistant PV (Vannucchi et al., 2015b; Passamonti et al., 2017), followed by approval of the JAK2 specific inhibitor, fedratinib, for the treatment of MF in 2019 (Harrison et al., 2017; Mullally et al., 2020; Venugopal and Mascarenhas, 2020). Both ruxolitinib and fedratinib are classified as type-I JAK inhibitors, competitively binding within the ATP-binding site of JAK2 in the active (DFG-in) conformation (Figure 3A) (Leroy and Constantinescu, 2017). Ruxolitinib therapy can limit further bone marrow fibrosis in *JAK2* p. V617F-driven MF and PV (Verstovsek et al., 2017b; Kroger et al., 2021) and multiple studies have shown that ruxolitinib therapy correlates with improved overall survival (Verstovsek et al., 2012a; Vannucchi et al., 2015a; Bose and Verstovsek, 2020; Kroger et al., 2021). However, the significance of this survival benefit is debated due to statistical limitations of the pioneer COMFORT-1 (NCT00952289) and COMFORT-2 (NCT00934544) trials (Passamonti et al., 2015; Cervantes and Pereira, 2017). Despite these limitations, ruxolitinib therapy significantly reduces splenomegaly, which is known to correlate with improved overall survival and can also improve patients’ quality of life (Verstovsek et al., 2012b; Vannucchi et al., 2015a; Harrison et al., 2016; Verstovsek et al., 2017a; Cervantes and Pereira, 2017; Bose and Verstovsek, 2020). Sustained symptomatic reductions have also been reported in MF patients who remain on long-term ruxolitinib therapy (Harrison et al., 2016; Verstovsek et al., 2017b).

Unfortunately, the use of ruxolitinib and fedratinib in MF has revealed a number of issues related to JAK2 as a therapeutic target and the consequences of type-I JAK2 inhibition. Ruxolitinib does not significantly reduce the mutant allele frequency. In a study by Deininger *et al.* (2015), ruxolitinib treatment reduced the *JAK2* p. V617F allele burden by >50% in only 12% of 236 MF patients (Deininger et al., 2015). Several studies have also reported a lack of significant spleen responses in a proportion of patients, where there was a less than 35% reduction in spleen volume (Harrison et al., 2012; Harrison et al., 2016; Verstovsek et al., 2017b; Gupta et al., 2020; Palandri et al., 2020). Furthermore, the majority of ruxolitinib-treated MF patients discontinue therapy due to dose-dependent adverse events, including thrombocytopenia and anemia (Harrison et al., 2016; Kuykendall et al., 2018; Bewersdorf et al., 2019; Palandri et al., 2020). The toxicity associated with ruxolitinib may be due to suppression of other JAK family kinases, with 6-fold selectivity over TYK2 and 130-fold selectivity over JAK3 (Quintas-Cardama et al., 2010). In addition, treatment discontinuation has been associated with severe ruxolitinib discontinuation syndrome, which is most likely caused by a rebound cytokine storm driven by the sudden release of accumulated phosphorylated JAK2 (pJAK2) (Coltro et al., 2017; Tvorogov et al., 2018; Palandri et al., 2021; Ross et al., 2021). Tvorogov *et al.* (2018) suggested that ruxolitinib binding promotes pJAK2 accumulation by preventing JAK2 dephosphorylation and degradation (Tvorogov et al., 2018; Ross et al., 2021). Despite the dose-dependent toxicity, low efficacy and the withdrawal syndrome associated with ruxolitinib therapy in MPNs, ruxolitinib remains the best available therapy (BAT) for MF and therefore, may be beneficial for ALL patients harboring *JAK2* alterations.

### Resistance to JAK2 Inhibitors

Introduction of TKIs into front-line combination therapies for Ph+ ALL has improved long-term outcomes primarily by improving complete remission rates, enabling more patients to become eligible for Allo-SCT (Bassan et al., 2010; Brissot et al., 2015; Chalandon et al., 2015). Therefore, despite the ongoing clinical challenges associated with JAK2 inhibition in the setting of MPNs, JAK2 inhibition may still reduce symptomatic burden of *JAK2*-altered ALL and improve outcomes by bridging more patients to Allo-SCT. However, the development of treatment resistance to kinase inhibitors is, unfortunately, a well-established occurrence following long-term targeted therapy in both hematologic malignancies and solid tumors (Gross et al., 2015; Bhullar et al., 2018; Pottier et al., 2020). The majority of Ph+ ALL patients treated with TKI who do not undergo Allo-SCT will ultimately relapse (Bassan et al., 2010; Fielding et al., 2014; Chalandon et al., 2015). Approximately 70–80% of Ph+ ALL patients who relapse following imatinib therapy harbor emergent mutations within the region encoding the ABL1 kinase domain of BCR:ABL1 (Pfeifer et al., 2007; Pfeifer et al., 2012; Soverini et al., 2014). Most imatinib-resistant mutations retain sensitivity to second-generation TKIs, including dasatinib, nilotinib and bosutinib, however resistance to these inhibitors can also occur via mutations such as *ABL1* p. T315I (Hochhaus et al., 2020). Similar to TKIs, all clinically available JAK2 inhibitors are ATP mimetics and there are concerns that incorporation of JAK2 inhibitors into treatment approaches for *JAK2*r ALL will lead to the development of resistance (Miller et al., 2014; Meyer, 2017).

The majority of ruxolitinib-treated MF patients lose their response over time, with a 3-years median duration of response (Harrison et al., 2016; Verstovsek et al., 2017b). The emergence of ruxolitinib-resistant mutations was initially suspected to underlie relapse of MF these patients, similar to the emergence of resistant mutations in TKI-treated CML and Ph+ ALL. JAK2 inhibitor-resistant mutations within *JAK2* have been identified primarily through *in vitro* random mutagenesis screens of *JAK2* (Hornakova et al., 2011; Deshpande et al., 2012; Weigert et al., 2012; Kesarwani et al., 2015; Wu et al., 2015). Screens performed *in vitro* by Kesarwani *et al.* (2015) identified 39 different *JAK2* mutations spanning across all domains of *JAK2* (FERM, SH2, pseudokinase, and kinase) that conferred resistance to ruxolitinib (Kesarwani et al., 2015). The *JAK2* p. Y931C mutation, homologous to the activating *JAK1* p. F958C mutation, was the first *JAK2* mutation identified to confer resistance to ruxolitinib and has been detected by *in vitro* screens from multiple groups (Hornakova et al., 2011). Several other *JAK2* mutations that confer resistance to ruxolitinib have been identified by saturation mutagenesis screens using cell lines expressing *JAK2* p. V617F or *CRLF2*/*JAK2* p. R683F (Deshpande et al., 2012; Weigert et al., 2012). All ruxolitinib-resistant *JAK2* mutations localize to the ATP/ruxolitinib binding site of the JAK2 kinase domain and confer cross-resistance to multiple type-I JAK inhibitors, suggesting that the ATP/ruxolitinib binding site is susceptible to JAK inhibitor-resistant mutations (Deshpande et al., 2012; Weigert et al., 2012; Downes et al., 2021).

However, despite *in vitro* predictions, clinical resistance to ruxolitinib in MF has not been reported to associate with any *JAK2* point mutations. This may be due to an insufficient selective pressure related to the low specificity and high toxicity of ruxolitinib (Downes et al., 2021; Ross et al., 2021). The absence of any *JAK2* point mutations in MF patients who acquired resistance to ruxolitinib suggests a role for a mutation-independent mechanism that enables persistent JAK/STAT signaling in the setting of long-term JAK2 inhibition (Koppikar et al., 2012; Harrison et al., 2020a; Ross et al., 2021). Ruxolitinib resistance in MF has been modelled *in vitro* by culturing cell lines expressing *JAK2* p. V617F long-term with ruxolitinib and demonstrated that ruxolitinib resistance occurs due to heterodimeric activation of *JAK2* p. V617F pJAK2 by other JAK family members, a mechanism now known as ruxolitinib persistence (Andraos et al., 2012; Koppikar et al., 2012; Tvorogov et al., 2018). Interestingly, ruxolitinib persistent cells could be re-sensitized following ruxolitinib withdrawal (Koppikar et al., 2012), consistent with a number of clinical reports following ruxolitinib rechallenging (Gisslinger et al., 2014; Gerds et al., 2018). However, this ruxolitinib persistence mechanism is not predicted to occur in *JAK2*r ALL as a recent study modelling acquired ruxolitinib resistance in *JAK2*r ALL *in vitro* identified emergent JAK inhibitor-resistant *JAK2* point mutations (Downes et al., 2021). Interestingly, one of these acquired mutations, *JAK2* p. G993A, also conferred resistance to the type-II JAK inhibitor, CHZ-868 (Downes et al., 2021). However, ruxolitinib resistance has not yet been reported in any ongoing clinical trials for ALL. There has only been one report of primary B-ALL leukemia cells harboring a *JAK2* kinase domain mutation and these cells demonstrated a reduced sensitivity to ruxolitinib (Sadras et al., 2017).

## Progress of Targeted Therapies for *JAK2*-Altered ALL

There were high expectations for ruxolitinib following its FDA-approval for MPNs in 2011 but unfortunately, ruxolitinib therapy has not matched the success of TKIs for CML. Consistent with reports of adverse events, JAK2 is a difficult protein to potently inhibit without toxic side effects as it plays an essential role in several normal cellular functions, including hematopoiesis (Levine et al., 2007; Vainchenker and Constantinescu, 2013; Akada et al., 2014). *JAK2* conditional knockout mice display severely impaired erythropoiesis (Akada et al., 2014; Grisouard et al., 2014; Fasouli and Katsantoni, 2021), whereas the myeloid-erythroid system of *ABL1* knockout mice appears normal (Hardin et al., 1995; Walz et al., 2008). All JAK2 inhibitors currently in development also target the JAK2 ATP-binding site, which is highly conserved across the JAK family and other kinases (Lucet et al., 2006; Singer et al., 2019). Imatinib also binds within the highly conserved ATP-binding site of ABL1, however, ruxolitinib inhibits a significantly higher number of kinases compared to imatinib (Davis et al., 2011), which may contribute to ruxolitinib’s increased treatment-related toxicity. Furthermore, clinical resistance to ruxolitinib occurs primarily through heterodimeric activation (Andraos et al., 2012; Koppikar et al., 2012; Tvorogov et al., 2018), rather than the emergence of point mutations, enabling therapeutic resistance despite ruxolitinib binding to WT and/or *JAK2* p. V617F-mutant JAK2. The adverse events associated with ruxolitinib therapy in MPNs suggests that similar clinical challenges will be observed when incorporating ruxolitinib into treatment approaches for *JAK2*-altered ALL.

Despite these limitations, the efficacy of JAK2 inhibitors has been demonstrated in several pre-clinical models of *JAK2*-mutant (Bercovich et al., 2008; Mullighan et al., 2009c; Yoda et al., 2010; Tasian et al., 2012; Van Bodegom et al., 2012; Wu et al., 2015; Steeghs et al., 2017) and *JAK2*r (Maude et al., 2012; Chase et al., 2013; Boer and den Boer, 2017; Downes et al., 2021) ALL. Type-I JAK2 inhibitors have been demonstrated to reduce cell proliferation and STAT5 phosphorylation in cell lines co-expressing *JAK2* p. R683 mutations and either *EPOR* or *CRLF2* (Bercovich et al., 2008; Mullighan et al., 2009c; Yoda et al., 2010; Tasian et al., 2012). However, there have been limited *ex vivo* studies assessing the efficacy of JAK2 inhibition in primary *CRLF2*r/*JAK2*-mutant ALL cells. Importantly, Steeghs *et al.* (2017) demonstrated that the *ex vivo* efficacy of ruxolitinib in *CRLF2*r/*JAK2*-mutant primary ALL cells was highly dependent on the addition of human TSLP (Steeghs et al., 2017). Human TSLPR cannot be activated by mouse TSLP (Van Bodegom et al., 2012; Francis et al., 2016) yet despite this dependence, patient-derived xenograft (PDX) models of *CRLF2*r/*JAK2*-mutant ALL cells have been generated in NSG mice (Maude et al., 2012; Suryani et al., 2015; Tasian SK. et al., 2017). This suggests the activation of alternative signaling pathways, such as RAS/MAPK, PI3K/PKB and mTOR, arguing against JAK2 inhibition as a precision medicine strategy for in *CRLF2*r/*JAK2*-mutant ALL (Winter et al., 2014; Tasian SK. et al., 2017; Steeghs et al., 2017). Furthermore, the dependence of *JAK2* mutations on human TSLPR activation suggests that conventional patient-derived xenograft (PDX) models of *CRLF2*r/*JAK2*-mutant ALL are not suitable to assess the efficacy of JAK2 inhibition (Francis et al., 2016; Steeghs et al., 2017; Kim et al., 2018). Consistent with this principle, ruxolitinib has only exhibited a low efficacy in PDX models of *CRLF2*r/*JAK2*-mutant ALL, despite reductions in peripheral blood and splenic blast counts (Maude et al., 2012). *CRLF2*r/*JAK2*-mutant PDX models engineered by Francis *et al.* (2016) to produce human TSLP may prove to be more clinically relevant models, enabling the *in vivo* efficacy of JAK2 inhibition for *CRLF2*r/*JAK2*-mutant ALL to be determined (Francis et al., 2016).

In contrast to *CRLF2*r/*JAK2* mutant primary cells, Roberts *et al.* (2014b) and Steeghs *et al.* (2017) have demonstrated that ruxolitinib treatment of *JAK2*r primary leukemic cells can significantly reduce cell viability and STAT5 phosphorylation (Roberts et al., 2014a; Steeghs et al., 2017). Similar results were also shown using murine B-cells transduced to express *JAK2* fusions (*BCR::JAK2*, *ETV6::JAK2*, *PAX5::JAK2*, *GOLGA4::JAK2,* or *ATF7IP::JAK2*) (Cuesta-Domínguez et al., 2012; Marit et al., 2012; Chase et al., 2013; Schinnerl et al., 2015; Downes et al., 2021; Downes et al., 2022), and *PAX5::JAK2* expressing *Arf* ^−/−^ murine pre-B cell models (Roberts et al., 2014a). Unlike PDX models of *CRLF2*r/*JAK2*-mutant ALL, the efficacy of ruxolitinib has also been demonstrated *in vivo* using PDX models of *JAK2*r ALL, where ruxolitinib treatment reduced peripheral blood blast counts and tumor burden (Roberts et al., 2012; Roberts et al., 2014a; Roberts KG. et al., 2017). An additive effect was also observed when used in combination with dexamethasone (Roberts KG. et al., 2017). However, ruxolitinib treatment did not induce complete remission in PDX models of *JAK2*r ALL (Maude et al., 2012; Roberts et al., 2012; Roberts et al., 2014a; Roberts KG. et al., 2017). This suggests that JAK2 inhibition in combination with chemotherapy may improve outcomes for ALL patients harboring *JAK2*r (Downes et al., 2021). This also suggests JAK2 inhibitors may also be an effective precision medicine strategy for *CRLF2*r/*JAK2*-mutant ALL, but more clinically relevant *in vivo* models of *CRLF2*r/*JAK2*-mutant ALL, that include human TSLP, are required to assess their efficacy.

Ruxolitinib is the only JAK inhibitor known to be undergoing clinical assessment in an ALL setting. High clinical effectiveness of ruxolitinib in combination with multi-agent chemotherapy has been reported in only small number of patients with either *CRLF2*r/*JAK*-mutant or *JAK2*r ALL (Schrappe et al., 2012; Roberts et al., 2014a; Schwab et al., 2016; Mayfield et al., 2017; Ding et al., 2018; Chen X. et al., 2019; Chen et al., 2022; Rizzuto et al., 2022). A phase 2 clinical trial (NCT02723994) led by the Children’s Oncology Group is currently assessing ruxolitinib in combination with chemotherapy for the treatment of ALL patients harboring *CRLF2* and/or *JAK* pathway alterations (Senkevitch and Durum, 2017). Results from the phase I of this trial recently reported no dose-limiting toxicity up to 50 mg/m^2^ dosed day 1–14 of a 28 days cycle, as well as continuous dosing at 40 mg/m^2^ post-induction chemotherapy (Tasian et al., 2018). Ruxolitinib therapy was also well tolerated and induced morphologic remission in a case report of a child with chemo-resistant *JAK2*r ALL and induction failure (Ding et al., 2018; Tasian et al., 2018). A phase 3 clinical trial (NCT03117751) is now investigating ruxolitinib/chemotherapy combination in patients with JAK-STAT signaling activation (Harvey and Tasian, 2020). There are also a number of other phase 1/2 clinical trials (NCT02420717, NCT03571321) assessing ruxolitinib for the treatment of Ph-like ALL harboring JAK/STAT pathway alterations. Early findings suggest that JAK inhibitors in combination with chemotherapy may improve outcomes for patients with these high-risk ALL subtypes, but we await the results of ongoing trials.

### Additional/Alternate Type-I JAK2 Inhibitors in Clinical Development

The myelosuppression resulting from ruxolitinib treatment in MF suggests that this should also be expected when including ruxolitinib in ALL treatment regimens. To attempt to overcome these limitations, several other JAK2 inhibitors have been assessed in clinical trials, but most have been discontinued primarily due to toxicity (Sonbol et al., 2013; Bose and Verstovsek, 2017). Current clinical studies of JAK2 inhibitors and their specificities are shown in Table 4. All JAK2 inhibitors being assessed clinically are type-I inhibitors, targeting the ATP-binding site of JAK2 in the active conformation. As the ATP-binding site is highly conserved among kinases, off-target suppression of JAK1 has been proposed to contribute to the myelosuppression and opportunistic infections associated with ruxolitinib therapy (Singer et al., 2016). Fedratinib is the most selective JAK2 inhibitor currently available and is likely less immunosuppressive than ruxolitinib due to weaker inhibition of JAK1 (Mullally et al., 2020; Talpaz and Kiladjian, 2021). Fedratinib has been demonstrated to significantly reduce splenomegaly and symptom burden in patients with either intermediate- or high-risk MF (Pardanani et al., 2011; Pardanani et al., 2015; Harrison et al., 2017; Mullally et al., 2020; Talpaz and Kiladjian, 2021). It is recent FDA-approval may reveal whether more selective JAK2 inhibition improves therapy-associated thrombocytopenia and anemia (Talpaz and Kiladjian, 2021).

**TABLE 4:** Clinical studies of type-I JAK2 inhibitors in hematological malignancies and their specificities. Myelofibrosis (MF), polycythemia vera (PV), essential thrombocythemia (ET), chronic myeloid leukemia (CML), acute myeloid leukemia (AML), chronic lymphocytic leukemia (CLL), small lymphocytic leukemia (SLL), acute lymphoblastic leukemia (ALL). Adapted from Raivola et al. (2021)(Raivola et al., 2021) and Vainchenker et al. (2018)(Vainchenker et al., 2018).

Inhibitor	Selectivity	Off-target	Diseases	Clinical phase
**Ruxolitinib (INCB-018424)**	JAK1/2>		MF, PV	FDA-approved
	TYK2, JAK3		CML, AML, CLL, SLL, ALL	Phase 2/3
**Fedratinib (TG101348)**	JAK2	FLT3, BRD4	MF	FDA-approved
**Momelotinib (CYT-387)**	JAK1/2	ALK-2, TBK1 IKKε	MF	Phase 3
**Pacritinib (SB11518)**	JAK2>TYK2	FLT3	MF	Phase 3
**Lestaurtinib**	JAK2/3		AML, MF, PV, ET	Phase 2
**Gandotinib (LY2784544)**	JAK2>JAK1		PV, ET, MF	Phase 2
**Ilginatinib (INCB-039110)**	JAK2>		MF	Phase 2
	JAK1/3, TYK2			
**NS-018**	JAK2	Src	MF	Phase 2/3
**AZD1480**	JAK2>JAK1	Aurora A, FGFR1, FLT4	MF	Phase 1

Unfortunately, fedratinib has also been associated with dose-dependent thrombocytopenia and anemia, in addition to gastrointestinal adverse events (Pardanani et al., 2011; Mullally et al., 2020). Approval of fedratinib also includes a “black box warning” on the risk of serious and fatal Wernicke encephalopathy (WE), a neurodegenerative condition traditionally caused by thiamine deficiency (Bewersdorf et al., 2019; Mullally et al., 2020). Suspected treatment-associated cases of WE resulted in the FDA issuing a clinical hold on fedratinib between 2013-2017, however these cases were ultimately determined to not be caused by fedratinib therapy (Bewersdorf et al., 2019; Mullally et al., 2020). The thrombocytopenia and anemia associated with fedratinib may be related to its on-target inhibition of WT JAK2 and off-target inhibition of FMS-like tyrosine kinase 3 (FLT3) and bromodomain-containing protein 4 (BRD4) (Talpaz and Kiladjian, 2021). It is currently unknown whether fedratinib is more effective than ruxolitinib as a first-line treatment for MF and no clinical trials are currently planned to assess these inhibitors head-to-head. However, a phase 2 study evaluating the efficacy of fedratinib for the treatment of ruxolitinib relapsed, refractory, or intolerant MF demonstrated significant reductions in splenomegaly and symptomatic burden (Harrison et al., 2020b; Talpaz and Kiladjian, 2021). In addition, fedratinib has been shown to bind both the ATP-binding site and the less conserved substrate-binding site of JAK2, a site which may be less prone to acquiring inhibitor-resistant mutations (Kesarwani et al., 2015). The high specificity of fedratinib for JAK2 suggests that it may associate with less dose-limiting toxicity in comparison to ruxolitinib and therefore, may be a more efficacious JAK inhibitor for the treatment of *JAK2*-altered ALL when used in combination with chemotherapy.

Other type-I JAK2 inhibitors currently being assessed in phase 3 clinical trials include momelotinib and pacritinib. Momelotinib is also type-I JAK1/2 specific inhibitor, similar to ruxolitinib, that was expected to improve symptoms of therapy-induced anemia by also inhibiting activin A receptor type 1 (ACVR1) (Asshoff et al., 2017). Unfortunately, momelotinib was not found to be superior at reducing symptom burden compared with ruxolitinib and consistent with this lower efficacy, momelotinib therapy was associated with fewer reports of anemia (Mesa et al., 2017a; Harrison et al., 2018; Bassiony et al., 2020). Another type-I JAK inhibitor assessed in phase 3 clinical trials is pacritinib, a type-I JAK2/FLT3 inhibitor that, similar to fedratinib, does not inhibit JAK1 (Talpaz and Kiladjian, 2021). Pacritinib therapy was demonstrated to be superior to the BAT at reducing splenomegaly (Mesa et al., 2017b; Mascarenhas et al., 2018) and was mostly non-myelosuppressive, likely due to weaker inhibition of JAK1 (Singer et al., 2016; Mesa et al., 2017b; Talpaz and Kiladjian, 2021). Pacritinib was also well-tolerated in patients with severe thrombocytopenia, suggesting that pacritinib may be beneficial for patients with anemia (Gerds et al., 2019; Harrison et al., 2020a; Bassiony et al., 2020; Verstovsek et al., 2021). To note, the efficacy of momelotinib and pacritinib for patients previously treated with ruxolitinib has not been reported (Harrison et al., 2020a). Fedratinib and pacritinib may reduce the leukaemic burden of *JAK2*-alterated ALL with superior or equivalent efficacy to ruxolitinib, potential improving complete remission rates and associating with less treatment-associated adverse events.

### Type-II JAK Inhibitors

In contrast to type-I JAK inhibitors, type-II JAK inhibitors bind the ATP-binding site of JAK2 in the inactive (DFG-out) conformation (Figure 3B) (Leroy and Constantinescu, 2017). Although type-II JAK inhibitors are still ATP-competitive, they are more specific for JAK2 by also binding a less conserved allosteric pocket, potentially minimizing toxicity by reduced inhibition of other kinases (Li et al., 2019). This type-II binding mode is similar to the inhibition of BCR:ABL1 with imatinib (Druker and Lydon, 2000; Schindler et al., 2000). The first type-II JAK inhibitor identified was BBT594 (Figure 3C), which was originally designed to inhibit BCR:ABL1 harboring the TKI-resistant *ABL1* p. T315I-mutation (Andraos et al., 2012). BBT594 inhibited STAT5 phosphorylation in cell models expressing either *TEL::JAK2* or *JAK2* p. V617F-mutant *JAK2*, albeit with low specificity and limited potency (Andraos et al., 2012). These findings prompted the development of the only other type-II JAK inhibitor, CHZ868 (Meyer et al., 2015; Li et al., 2019). CHZ868 potently inhibited JAK2 with a high selectivity over other JAK family members (Meyer et al., 2015; Wu et al., 2015). Promising pre-clinical studies showed that CHZ868 not only improved survival and leukaemic burden in *vivo* models of MPN and B-ALL, but preferentially inhibited *JAK2* p. V617F-mutant *JAK2* hematopoietic cells over WT *JAK2* cells (Meyer et al., 2015; Wu et al., 2015). CHZ868 also reduced the *JAK2* p. V617F allele burden in these MPN models, which is not observed with type-I inhibitor treatment (Meyer et al., 2015). The potent activity of type-II inhibitors against *JAK2* p. V617F-mutant hematopoietic cells suggests that they may be a more effective than type-I JAK inhibitors for the treatment of *JAK2*-altered ALL.

By binding the inactive conformation of JAK2, type-II JAK inhibitors may also prevent the development of resistance through the persistent JAK/STAT signaling that is associated with ruxolitinib therapy. Type-II JAK inhibitors have been demonstrated to reduce the proliferation of ruxolitinib-persistent cell models and inhibit JAK2 activation loop phosphorylation (Andraos et al., 2012; Koppikar et al., 2012; Meyer et al., 2015; Tvorogov et al., 2018). The inhibition of JAK2 activation loop phosphorylation by CHZ868 has been suggested to prevent accumulation of pJAK2, preventing heterodimeric JAK2 activation and subsequent inhibitor persistence (Meyer et al., 2015; Tvorogov et al., 2018). CHZ868 did not facilitate the accumulation of phosphorylated JAK2 in hematopoietic cell lines or primary *JAK2* p. V617F cells (Tvorogov et al., 2018). Furthermore, CHZ868 withdrawal was not associated with a rebound in STAT5 signaling (Tvorogov et al., 2018), suggesting that type-II JAK2 inhibition may not be associated with withdrawal syndrome (Meyer et al., 2015; Wu et al., 2015; Tvorogov et al., 2018). However, one *JAK2* mutation (*JAK2* p. L884P) confers resistance to both BBT594 and CHZ868, suggesting that resistance to type-II JAK inhibitors may still occur through mutations within the *JAK2* ATP-binding site (Wu et al., 2015; Leroy and Constantinescu, 2017). Unfortunately, the potent activity of type-II JAK inhibitors also risks stronger suppression of normal hematopoiesis through greater inhibition of WT JAK2, but without clinical assessment it is unknown whether type-II JAK inhibitors would result in more or less pronounced cytopenia (Vainchenker et al., 2018; Ross et al., 2021). The risk of severe cytopenia may underlie why neither CHZ868 nor BBT594 were pursued for further drug development (Andraos et al., 2012; Wu et al., 2015). Further research is needed to determine whether type-II JAK inhibitors are clinically viable and their susceptibility to persistence or resistance. The development of type-II JAK inhibitors may be an effective therapeutic approach for *JAK2*-altered ALL, enabling JAK2 inhibition without risking the withdrawal syndrome and disease persistence that is associated with type-I JAK inhibitors.

### Allosteric JAK Inhibitors (Type-III JAK Inhibitors)

Type-II JAK inhibitors still target the highly conserved ATP-binding site of JAK2, which may lead to toxicity due to off-target effects on other kinases. In contrast, allosteric JAK inhibitors, also referred to as type-III JAK inhibitors, bind less conserved allosteric pockets outside of the JAK2 ATP-binding site (Leroy and Constantinescu, 2017). In addition to the substrate binding site, three potentially targetable allosteric sites of the JAK2 kinase domain have been computationally identified, but inhibitors of these sites have not yet been verified (Kesarwani et al., 2015; Leroy and Constantinescu, 2017). These allosteric sites are less conserved than the ATP-binding site and therefore, may offer greater selectivity and potency compared with type-I or type-II JAK inhibitors (Kesarwani et al., 2015). Two non-ATP-competitive JAK inhibitors, ON044580 and LS104, have been described as allosteric JAK inhibitors and demonstrated efficacy *in vitro* against *JAK2* p. V617F transformed cell lines and primary patient cells (Lipka et al., 2008; Jatiani et al., 2010). The allosteric binding mechanisms of these compounds and their *in vivo* efficacy were never determined, although they did demonstrate substrate-competitive binding modes (Lipka et al., 2008; Jatiani et al., 2010; Raivola et al., 2021). The JAK2 substrate-binding site may be less susceptible to inhibitor-resistant mutations than other allosteric sites as mutations may prevent substrate binding, which is essential for the downstream signaling activation (Kesarwani et al., 2015). To support this hypothesis, no JAK inhibitor-resistant mutations have been identified within the substrate-binding site by random mutagenesis of JAK2 (Kesarwani et al., 2015). These findings suggest that the JAK2 substrate-binding site may be a novel and effective targetable site for *JAK2*-alterated malignancies, and long-term therapy may not result in the development of resistance (Kesarwani et al., 2015).

### Targeted JAK2 Degradation

Direct targeting of JAK2 by proteolysis-targeting chimeras (PROTACS) have recently emerged as an approach to limit withdrawal syndrome and ruxolitinib persistence, whilst still inhibiting signaling activation through JAK2 (Shah et al., 2020; Chang et al., 2021). PROTACS comprise three distinct components: a ligand for E3 ligase, a ligand for the protein of interest, and a linker to couple the two functional ligands (Kargbo, 2021). JAK2 PROTACs facilitate the formation of E3-PROTAC-JAK2 complexes, inducing E3 ligase-mediated ubiquitination and subsequent proteasomal degradation of JAK2 (Kargbo, 2021). Current JAK2 PROTACs have been designed using the full-length, or a portion, of known type-I JAK inhibitors as the JAK2-targeting ligand and have been shown to induce JAK2 ubiquitination and degradation in leukaemic cell lines (Shah et al., 2020; Chang et al., 2021; Kargbo, 2021). Strikingly, Chang *et al.* (2021) demonstrated significant reductions in leukaemic burden in *vivo CRLF2*r ALL models treated with JAK2 PROTACS, but not ruxolitinib monotherapy (Chang et al., 2021). The superior activity of these PROTACs was attributed to both JAK2 inhibition and targeted degradation of proteins including JAK1/2/3, TKY2, IKZF1/3, and G1 to S Phase Transition 1 (GSPT1) (Chang et al., 2021). Degradation of multiple targets with a single PROTAC establishes the basis for PROTACs with modifiable specificity and high efficacy in malignancies driven by JAK-STAT. However, this degradation of multiple targets may result in toxic side effects if PROTAC target proteins are required for hematopoiesis or B-cell maintenance. Furthermore, the clinical viability of JAK2 PROTACs is yet to be determined and, in the absence of PROTAC ligands specific for mutant-JAK2 or JAK2 fusions, JAK2 PROTACS may induce anemia and thrombocytopenia due to degradation of WT JAK2, therefore, further investigations are required.

Inhibition of heat shock protein 90 (HSP90) has also been explored as a less targeted approach to degrade JAK2 (Brkic and Meyer, 2021; Raivola et al., 2021). Degradation of JAK2 is reduced through stabilization by chaperones including HSP90 (Bose and Verstovsek, 2017; Ross et al., 2021). HSP90 inhibitors, such as PU-H71 and AUY922, degrade JAK2 and inhibit downstream signaling in cell lines expressing *JAK2* p. V617F (Marubayashi et al., 2010; Fiskus et al., 2011) and cells harboring JAK inhibitor-resistant mutations (Weigert et al., 2012). HSP90 inhibitor monotherapy (Marubayashi et al., 2010), or in combination with a JAK2 inhibitor (Fiskus et al., 2011; Bhagwat et al., 2014), also significantly reduced leukaemic burden in *JAK2* p. V617F murine models. Early phase clinical trials assessing PU-H71 (Speranza et al., 2018) and AUY922(Hobbs et al., 2018) demonstrated reductions in splenomegaly, however, both trials were terminated due to toxicity. Histone deacetylase (HDAC) inhibitors, such as panobinostat and vorinostat, have also been investigated as a strategy to inhibit HSP90 activity by promoting HSP90 hyperacetylation (Brkic and Meyer, 2021). The efficacy of HDAC inhibitors has been demonstrated in *vitro* and *in vivo* models of *JAK2* p. V617F-mutant MPN, particularly in combination with a JAK inhibitor (Wang et al., 2009; Akada et al., 2012; Evrot et al., 2013). However, HDAC inhibitors in combination with ruxolitinib were not superior to ruxolitinib alone in recent phase 1/2 MPN clinicals trials (Bose et al., 2019; Mascarenhas et al., 2020). Similar to AUY922, vorinostat was also associated with adverse effects (Andersen et al., 2013). This suggests that HSP90 inhibition may be too toxic to be clinically viable. However, the more tolerable HDAC inhibitor, givinostat, is expected to be assessed in a phase 3 clinical trial for the treatment of PV, suggesting that givinostat may be a potential therapeutic approach for the treatment of *JAK2*-altered ALL when in combination with chemotherapy or type-I JAK2 inhibition (Chifotides et al., 2020).

## Discussion - Future Outlooks

The *CRLF2*/*JAK2*-mutant and *JAK2*r subtypes of ALL correlate with poor prognosis and targeted JAK2 inhibition remains a feasible precision medicine approach. Inhibition of mutant-JAK2 or JAK2 fusions using targeted therapeutic strategies would abolish the resulting constitutively active JAK/STAT signaling. Such approaches may improve patient outcomes by increasing complete remission rates, enabling more patients to be eligible for allogeneic transplantation therapy. Current precision medicine approaches that are being investigated for the treatment of *JAK2*-altered ALL are shown in Table 5. To date, only type-I JAK inhibitors have been tested *in vivo* for the treatment of *JAK2*-altered ALL with most clinical data involving ruxolitinib thus far. Since the approval of ruxolitinib for the treatment of MF, development of other type-I JAK inhibitors has focused on reducing the treatment-related myelosuppression associated with ruxolitinib therapy. However, momelotinib has not demonstrated superior efficacy compared with ruxolitinib, and no clinical trials are planned to assess fedratinib or pacritinib against ruxolitinib head-to-head. Nonetheless, the recent FDA approval of fedratinib for the treatment of MPNs may, in future, give some indication as to whether highly specific JAK2 inhibition can improve treatment-related thrombocytopenia and anemia. Several studies have also assessed ruxolitinib combination therapies with other disease-modifying agents to improve side effects and therapeutic responses through synergistic activities (Böhm et al., 2021). The vast array of ruxolitinib combination therapies have been extensively reviewed by Kuykendall *et al.* (2020) and includes HDAC inhibitors, DNA methyltransferases inhibitors, erythropoiesis-stimulating agents, BCL-2 (B-cell lymphoma 2) inhibitors, BET (bromodomain and extra-terminal protein) inhibitors, and many others (Kuykendall et al., 2020).

**TABLE 5:** Current precision medicine approaches to target JAK2 in *JAK2*-altered ALL and their associated benefits and disadvantages.

Therapeutic strategy	Examples	Benefits	Disadvantages
**Type-I inhibitors**	Ruxolitinib	May reduce blast counts when in combination with chemotherapy	Withdrawal syndrome
	Fedratinib		Resistant/persistent disease
	Momelotinib		Cytopenia
	Pacritinib		
**Type-II inhibitors**	BBT594	High JAK2 specificity	Risk of severe cytopenia
	CHZ868	May not associate with withdrawal syndrome	Risk of resistant/persistent disease
			Development of both available inhibitors has terminated
**Allosteric inhibitors (type-III inhibitors)**	LS104	Lower risk of cytopenia	Binding mechanisms of available inhibitors unknown
	ON044580	May be less susceptible to resistant/persistent disease	Development of both available inhibitors has terminated
			Association with withdrawal syndrome unknown
**HSP90 inhibitors**	PU-H71	May reduce blast counts when in combination with type-I JAK inhibitors	Associated with severe adverse events
	AUY922	Degrades JAK2 harboring inhibitor-resistant mutations	Unknown risk of resistant disease
		Not associated with withdrawal syndrome	Development of both available inhibitors has terminated
**HDAC inhibitors**	Panobinostat	May reduce blast counts when in combination with type-I JAK inhibitors	May associate with severe adverse events
	Vorinostat	Degrades JAK2 harboring inhibitor-resistant mutations	Unknown risk of resistant disease
	Givinostat	Not associated with withdrawal syndrome	
**PROTACS**	-	Demonstrates superior efficacy than ruxolitinib *in vivo*	Risk of cytopenia and adverse events
		Should degrade JAK2 harboring inhibitor-resistant mutations	Unknown risk of resistant disease
		Not associated with withdrawal syndrome	

Type-II JAK inhibitors offer several potential advantages over type-I inhibition. Type-II JAK inhibitors offer the opportunity to specifically target JAK2 by binding an additional allosteric site, and the potential to prevent withdrawal syndrome by binding the inactive conformation of JAK2. Although clinical development of CHZ868 was not pursued, pre-clinical studies demonstrated promising results. Research and development should continue to determine the clinical viability of type-II JAK inhibitors. JAK2 PROTACs, which degrade ligand bound-JAK2, also offer another potentially efficacious approach and may prevent withdrawal syndrome. Recent research demonstrating the superior efficacy of JAK2 PROTACS over ruxolitinib in murine models suggests that JAK2 PROTACs may be an effective therapeutic strategy for the treatment of JAK2-altered malignancies, and future clinical evaluation is warranted. Allosteric inhibitors are also another possible approach to more specifically target JAK2 by binding regions that are less conserved among other kinases. The field of allosteric JAK inhibitors are still in their relative infancy but the discovery that fedratinib binds to both the ATP- and substrate-binding sites of JAK2 suggests that this is promising area for future development. Allosteric JAK inhibitors used in combination with type-I JAK inhibitors, or inhibitors targeting the substrate-binding site, may also impede the development of acquired resistance.

The ATP-binding site of the JAK2 pseudokinase domain could potentially be targetable with small molecular inhibitors to stabilize the JH2-JH1 autoinhibitory interaction. To support this hypothesis, targeting of the TYK2 pseudokinase domain has been demonstrated to inhibit TYK2 activation (Tokarski et al., 2015). However, it is unknown whether this approach is translatable to JAK2 as targeting this site may actually activate JAK2 by preventing *JAK2* p. S523 and p. Y570 phosphorylation, destabilizing the JH2-JH1 autoinhibitory interaction. No compounds have been identified to bind to the JAK2 pseudokinase domain ATP-binding site to date. In addition, point mutations within the JAK2 FERM domain have been demonstrated to abolish the JAK2 association with TPOR (Royer et al., 2005), indicating that the interface between the JAK2 FERM-SH2 domains and the cytoplasmic region of the associated cytokine receptors may be a novel targetable site. Inhibiting the association of JAK2 with cytokine receptors may prevent JAK2 dimerization and subsequent activation. This approach may also reduce toxic side effects by not only specifically targeting JAK2, but also specific receptors and their complexes with JAK2. Another precision medicine approach for *CRLF2/JAK2*-mutant ALL could also include strategies that inhibit TSLPR dimerization or activation (Markovic and Savvides, 2020) potentially by using antagonistic monoclonal antibodies (Zhang et al., 2011; Mohamed et al., 2021; Numazaki et al., 2021), inhibitors (Van Rompaey et al., 2017), or CAR T-cells (Qin et al., 2015). Therapeutic approaches that offer more selectivity for JAK2 should improve the toxic side effects associated with JAK2 inhibition. However, due to the inherent essential role of WT JAK2 in normal hematopoiesis, therapeutic approaches that do not offer selectivity for JAK2 alterations over WT JAK2 will likely associate with a risk of anemia and thrombocytopenia.

Future drug design approaches that are specific for mutant-JAK2 or JAK2 fusions would ultimately provide less toxic and more effective therapies for MPN and ALL patients harboring *JAK2* alterations. If *JAK2* mutations or *JAK2*r are overexpressed in ALL patients, there are potentially therapeutic strategies that can influence gene expression. One such approach may be through BET inhibitors, which have been shown to disrupt super-enhancers, promoter enhancers often associated with oncogenes (Crump et al., 2021). Additionally, the majority of *JAK2* mutations associated with ALL lie within *JAK2* exon 16, localizing to the ATP-binding site of the JAK2 pseudokinase domain. Several lines of evidence suggest that allosteric inhibitors could target the JAK2 pseudokinase domain ATP-binding site, and there is potential for these inhibitors to be designed to target specific JAK2 mutants. In particular, it has been postulated that *JAK2* p. R683 mutations disrupt the pseudokinase domain-mediated autoinhibitory interaction, therefore inhibitors that can stabilize this interaction may be able to overcome the effects of these mutations. The recent discovery that apposing JAK1 monomers dimerize via their pseudokinase domains also positions this dimerisation interface as a potentially novel targetable site (Glassman et al., 2022). JAK2 dimerisation may also be mediated via the pseudokinase domains however the full-length structure of JAK2 is yet to be determined. Future development of JAK2 inhibitors that aim to either stabilize the pseudokinase domain-mediated autoinhibitory interaction, or hinder pseudokinase-domain mediated dimerisation, may prove to be effective new therapeutic approaches to target JAK2. Importantly, targeting JAK2 dimerisation, rather than its activation, through such precision medicine strategies may prevent JAK2 heterodimerization as a mechanism of drug resistance.

Therapeutic targeting of the JAK2 pseudokinase domain also offers opportunities to target JAK2 proteins that harbor specific mutations within this region. The molecular activation mechanisms of ALL-associated *JAK2* p. R683 mutations are yet to be fully elucidated but further structural research may inform future drug design strategies to target *JAK2* p. R683-mutant JAK2. However, in contrast to JAK2 mutations, the majority of JAK2 fusions do not retain the ATP-binding site of the JAK2 pseudokinase domain (encoded by *JAK2* exons 13–15). This restricts targetable regions within the JAK2 portion of the fusion to the kinase domain. Therapeutic approaches that target the JAK2 kinase domain will not have the ability to distinguish between JAK2 fusions and WT JAK2, preventing potent inhibition of leukaemic cell growth without toxic side effects. Future work to elucidate the structure of different JAK2 fusions may potentially reveal novel targetable sites that are specific for JAK2 fusions over WT JAK2. A deeper understanding of the cytokine-independent oligomerization mechanism that is hypothesized to underlie the constitutive activation of JAK2 fusions may also reveal novel targetable sites. In particular, there may be similar oligomerization domains within the JAK2 fusion partners, such as the CC and HLH motifs. Inhibitors that can bind these motifs and prevent JAK2 fusion dimerization may be a potential therapeutic strategy to achieve specificity for JAK2 fusions and reduce the toxic side effects observed with traditional JAK2 inhibition.

Ultimately, future approaches targeting JAK2 need to 1) target less conserved regions to achieve a higher specificity for JAK2 over other JAK family members, 2) avoid other kinases with potential for off-target toxicity, 3) inhibit inactive rather that active conformation and 4) target mutant forms of JAK vs. WT using allosteric or novel domain-domain inhibition. Combination strategies using type-I JAK inhibitors or PROTACs may also offer improved efficacy over ruxolitinib. Future research into these therapeutic approaches, and the design of inhibitors targeting mutant-JAK2 and JAK2-fusions, is urgently needed to improve outcomes for the high-risk *JAK2*-altered subtypes of ALL.

## Glossary

ABL1: Abelson 1
ACVR1: Activin A Receptor Type 1
ALK: ALK Receptor Tyrosine Kinase
Allo-SCT: Allogenic Stem Cell Transplantation
ATF7IP: Activating Transcription Factor 7 Interacting Protein
ATP: Adenosine Triphosphate
ALL: Acute Lymphoblastic Leukemia
B-ALL: B-cell Acute Lymphoblastic Leukemia
BAT: Best Available Therapy
BCL-2: B-cell Lymphoma 2
BCR: Breakpoint Cluster Region Protein
BET: Bromodomain and Extra-terminal Protein
BRD4: Bromodomain-Containing Protein 4
BTB: BR-C, ttk and bab
BTG1: BTG Anti-Proliferation Factor 1
BTLA: B and T Lymphocyte Associated
CAR: Chimeric Antigen Receptor
CDKN2: Cyclin-Dependent Kinase Inhibitor 2
CD200: CD200 Molecule
CML: Chronic Myeloid Leukemia
C-Terminus: Carboxyl Terminus
CC: Coiled Coil
CRLF2: Cytokine Receptor-Like Factor 2
CRLF2r: CRLF2-Rearrangment/Rearranged
DS-ALL: Down-Syndrome Acute Lymphoblastic Leukemia
EBF1: EBF Transcription Factor 1
ET: Essential Thrombocythemia
EPOR: Erythropoietin Receptor
ETV6: ETS Variant Transcription Factor 6
FERM: 4.1 Protein, Ezrin, Radixin, Moesin
FGFR1: Fibroblast Growth Factor Receptor 1
FLT3: FMS-like Tyrosine Kinase 3
FOP: FGFR1 Oncogene Partner
GAP: GTPase Activating Protein
GAS: Gamma-Activated Site
GEF: Guanidine Nucleotide Exchange factor
GHR: GH Receptor
GOLGA: Golgin Subfamily A
GSPT1: G1 to S Phase Transition 1
HDAC: Histone Deacetylase
hGHR: Human Growth Hormone Receptor
HLH: Helix-Loop-Helix
HMBOX1: Homeobox Containing 1
HR: High-Risk
HSC: Hematopoietic Stem Cell
HSP: Heat Shock Protein
IL-7Rα: Interleukin 7 Receptor Alpha Chain
IKZF1: IKAROS Family Zinc Finger 1
JAK: Janus Kinase
JAK2: Janus Kinase 2
JAK2r: JAK2-Rearrangment/Rearranged
JH: JAK Homology
KIR: Kinase Inhibitory Region
KRAS: KRAS Proto-Oncogene
LisH: LIS1 Homology
LNK: Lymphocyte Adaptor Protein
MAPK: Mitogen-Activated Protein Kinase
MF: Myelofibrosis
MPN: Myeloproliferative Neoplasms
MPRIP: Myosin Phosphatase Rho Interacting Protein
mTOR: Mammalian Target Of Rapamycin
NTRK3: Neurotrophic Receptor Tyrosine Kinase 3
NPC: Nuclear Pore Complexes
N-terminus: Amino Terminus
NLS: Nuclear Localization Signal
OFD1: OFD1 Centriole and Centriolar Satellite Protein
PAX5: Paired Box 5
PCM1: Pericentriolar Material 1
PDGFRA: Platelet-Derived Growth Factor Receptor A
PDX: Patient-Derived Xenograft
PI3K: Phosphatidylinositol-4,5-Bisphosphate 3-Kinase: Phosphatidylinositol-4,5-Bisphosphate 3-Kinase
Ph+ ALL: Ph-positive Acute Lymphoblastic Leukemia
Ph-like ALL: Ph-like Acute Lymphoblastic Leukemia
PI3K: Phosphatidylinositol-4,5-Bisphosphate 3-Kinase: Phosphatidylinositol-4,5-Bisphosphate 3-Kinase
PKB: Protein Kinase B
PMF: Primary Myelofibrosis
PNT: Pointed Domain
PPFIBP: PTPRF Interacting Protein
PROTACS: Proteolysis-Targeting Chimeras
PTP: Protein Tyrosine Phosphatase
PTPN: Protein Tyrosine Phosphatase Non-Receptor
PTPR: Protein Tyrosine Phosphatase Receptor
PV: Polycythemia Vera
RAG: Recombination-Activating Gene
RAS: RAS GFP-Activating Protein
RBX2: RING-Finger-Domain-Only Protein
RFX3: Regulatory Factor X3
RNPC3: RNA Binding Region Containing 3
ROCK: Rho Associated Coiled-Coil Containing Protein Kinase
RUNX1: RUNX Family Transcription Factor 1
SAM: Sterile Alpha Motif
SETD1: SET Domain-Containing 1A
SH2: Src Homology 2
SH2L: SH2-like
SLL: Small Lymphocytic Leukemia
SMU1: SMU1 DNA Replication Regulator and Spliceosomal Factor
SNX29: Sorting Nexin 29
SOCS: Suppressor Of Cytokine Signaling
SPAG9: Sperm Associated Antigen 9
SR: Standard Risk
SSBP2: Single Stranded DNA Binding Protein 2
STAT: Signal Transducer and Activator of Transcription
STRBP: Spermatid Perinuclear RNA Binding Protein
STRN3: Striatin 3
T-ALL: T-cell Acute Lymphoblastic Leukemia
TBL1XR1: TBL1Z Receptor
TERF2: Telomeric Repeat Binding Factor 2
TKI: Tyrosine Kinase Inhibitor
TPM3: Tropomyosin 3
TPOR: Thrombopoietin Receptor
TPR: Translocated Promoter Region
TSLPR: Thymic Stromal Lymphopoietin Receptor
TYK2: Tyrosine Kinase 2
USP25: Ubiquitin Specific Peptidase 25
VPREB: V-set Pre-B Cell Surrogate Light Chain 1
WE: Wernicke Encephalopathy
WT: Wild-Type
ZBTB: Zinc Finger and BTB Domain-Containing
ZEB2: Zinc Finger E-box Binding Homeobox 2
ZMYM2: Zinc Finger MYM-type Protein 2
ZNF: Zinc Finger Protein
