# Supplementary material for: JAK2 Alterations in Acute Lymphoblastic Leukemia: Molecular Insights for Superior Precision Medicine Strategies
Source: Front Cell Dev Biol. 2022 Jul 12;10:942053. doi: 10.3389/fcell.2022.942053 (PMC9315936; doi:10.3389/fcell.2022.942053)
Supplement: Supplementary file 1 [file DataSheet1.PDF]

Supplementary Material

Supplementary Table I. All reported *JAK2* fusion genes in patients with *JAK2*-rearranged acute lymphoblastic leukemia. To date, 94 patients have been diagnosed with *JAK2*-rearranged acute lymphoblastic leukemia and a total of 26 fusion partners have been identified.

Fusion Partner	*JAK2* Translocation	Breakpoint within *JAK2*	Breakpoint within Fusion	Patient Sex/ Age (year)	T-ALL/ B-ALL	*IKZF1* Alteration	CR/ relapse	Reference
**ATF7IP (12p13.1)**	NA[a]	exon 17	exon 13	M/10-15	B-ALL	Yes	NA	(Roberts et al., 2014)
	NA[a]	exon 16	exon 13	M/40-59	B-ALL	Yes	NA	(Roberts et al., 2017)
	NA[a]	exon 8	exon 10	F/40-59	B-ALL	No	NA	(Roberts et al., 2017)
**BCR (22q11.2)**	t(9;22)(p24.1;q11.2)	NA	NA	M/14	B-ALL	NA	Yes/No	(Tirado et al., 2010)
	t(3;22;9)(p12;q11.2;p24)	exon 15	exon 1	M/2.7	B-ALL	Yes	NA	(Roberts et al., 2012)
	t(9;22)(p24.1;q11.2)	NA	NA	M/13	B-ALL	NA	Yes/No	(Tirado et al., 2013)
	NA[a]	exon 19	exon 1	M/21-39	B-ALL	Yes	NA	(Roberts et al., 2014), (Roberts et al., 2017)
	NA[a]	exon 15 & 17	exon1	M/10-15	B-ALL	Yes	NA	(Roberts et al., 2014)
	t(9;22;15)(p24;q11;q21)	exon 17	exon 1	M/58	B-ALL	Yes	Yes/No	(Duployez et al., 2016)
	NA[a]	exon 17	exon 1	NA	B-ALL	Yes	Yes/Yes	(Boer et al., 2017)
	NA[a]	NA	NA	NA	B-ALL	NA	NA	(Reshmi et al., 2017)
	NA[a]	NA	NA	NA	B-ALL	NA	NA	(Reshmi et al., 2017)
	NA[a]	NA	NA	M/NA	B-ALL	NA	NA	(Reshmi et al., 2017)
	NA[a]	exon 17	exon 1	NA/5	B-ALL	NA	Yes/Yes	(Steeghs et al., 2017)
	NA[a]	NA	NA	NA	B-ALL	NA	NA	(Chen et al., 2021)
**EBF1 (5q33.3)**	NA[a]	exon 17	exon 14	F/16-20	B-ALL	Yes	NA	(Roberts et al., 2014)
**ERC1 (12p13.33)**	NA[a]	NA	NA	NA	B-ALL	NA	NA	(Chen et al., 2021)
**ETV6 (12p13)**	t(9;12)(p24;p13)	exon 17	exon 4	M/1.6	B-ALL	NA	Yes/Yes	(Peeters et al., 1997)
	t(9;12)(p24;p13)	exon 19	exon 5	M/4	T-ALL	NA	Yes/Yes	(Lacronique et al., 1997)
	t(9;12)(p24;p13)	NA[b]	NA[b]	F/25	T-ALL	NA	No	(Zhou et al., 2012)
	t(9;12)(p24;p13)	NA[b]	NA[b]	M/32	B-ALL	NA	No	(Zhou et al., 2012)
	t(9;12)(p24;p13)	NA[b]	NA[b]	M/42	B-ALL	NA	No	(Zhou et al., 2012)

	NA[a]	exon 17	exon 4	M/26.4	B-ALL	Yes	NA	(Roberts et al., 2014), (Roberts et al., 2017)
	t(9;12)(p24;p13)	exon 17	exon 5	NA/2.6	B-ALL	NA	NA	(Roberts et al., 2014)
	NA[a]	NA	NA	M/NA	B-ALL	NA	NA	(Reshmi et al., 2017)
	NA[a]	NA	NA	M/NA	B-ALL	NA	NA	(Roberts et al., 2018)
**GOLGA4 (3p22.2)**	NA[a]	exon 19	exon 11	M/54	B-ALL	Yes	Yes/No	(Downes et al., 2021)
**GOLGA5 (14q32)**	NA[a]	exon 12	exon 10	M/10	B-ALL	Yes	Yes/No	(Ding et al., 2018)
**HMBOX1 (8p21.1-p12)**	NA[a]	NA	NA	NA	B-ALL	NA	NA	(Tasian et al., 2017)
**MPRIP (17p11.2)**	t(9;17)(p24.1;q11.2)	NA	NA	M/NA	B-ALL	NA	NA	(Grioni et al., 2019)
**NPHP3 (3q22.1)**	NA[a]	NA	NA	NA	B-ALL	NA	NA	(Chen et al., 2021)
**OFDI (Xp22.2)**	NA[a]	exon 13	exon 22	M/11	B-ALL	Yes	Yes/No	(Yano et al., 2015)
	NA[a]	exon 13	exon 22	M/3	B-ALL	Yes	No	(Imamura et al., 2016)
**PAX5 (9p13.2)**	NA[c]	exon 18	exon 5	NA/9.6	B-ALL	NA	Yes/Yes	(Nebral et al., 2009)
	NA[a]	exon 19	exon 6	NA/6.9	B-ALL	NA	Yes/No	(Nebral et al., 2009)
	NA[a]	exon 19	exon 5	M/14	B-ALL	NA	NA	(Coyaud et al., 2010)
	NA[a]	exon 19	exon 5	F/12.9	B-ALL	Yes	NA	(Roberts et al., 2012)
	NA[a]	exon 19	exon 5	M/21-39	B-ALL	Yes	NA	(Roberts et al., 2014), (Roberts et al., 2017)
	NA[a]	exon 19	exon 5	M/21-39	B-ALL	Yes	NA	(Roberts et al., 2014), (Roberts et al., 2017)
	NA[a]	exon 19	exon 5	F/10-15	B-ALL	Yes	NA	(Roberts et al., 2014)
	NA[a]	exon 19	exon 5	NA/10-15	B-ALL	Yes	NA	(Roberts et al., 2014)
	NA[a]	exon 19	exon 5	F/16-20	B-ALL	No	NA	(Roberts et al., 2014)
	NA[a]	exon 19	exon 5	M/16-20	B-ALL	Yes	NA	(Roberts et al., 2014)
	NA[a]	exon 19	exon 5	F/5.3	B-ALL	NA	NA	(Roberts et al., 2014), (Reshmi et al., 2017)
	NA[a]	NA	NA	F/10	B-ALL	Yes	Yes/Yes	(Imamura et al., 2016)
	NA[a]	exon 19	exon 5	F/NA	B-ALL	Yes	Yes/No	(Boer et al., 2017)
	NA[a]	exon 19	exon 5	F/NA	B-ALL	No	Yes/No	(Boer et al., 2017)

	NA[a]	exon 19	exon 5	M/NA	B-ALL	Yes	Yes/No	(Boer et al., 2017)
	NA[a]	exon 19	exon 5	F/40-59	B-ALL	Yes	NA	(Roberts et al., 2017)
	NA[a]	exon 19	exon 7	F/40-59	B-ALL	Yes	NA	(Roberts et al., 2017)
	NA[a]	exon 18	exon 5	F/60-86	B-ALL	No	NA	(Roberts et al., 2017)
	NA[a]	NA	NA	NA	B-ALL	NA	NA	(Reshmi et al., 2017)
	NA[a]	NA	NA	NA	B-ALL	NA	NA	(Reshmi et al., 2017)
	NA[a]	NA	NA	NA	B-ALL	NA	NA	(Reshmi et al., 2017)
	NA[a]	exon 19	exon 5	NA/1	B-ALL	NA	Yes/No	(Steeghs et al., 2017)
	NA[a]	exon 19	exon 5	NA/14	B-ALL	NA	Yes/No	(Steeghs et al., 2017)
	NA[a]	exon 19	exon 5	NA/1	B-ALL	NA	Yes/No	(Steeghs et al., 2017)
	NA[a]	NA	NA	M/NA	B-ALL	NA	NA	(Roberts et al., 2018)
	t(9;9)	NA	NA	M/NA	B-ALL	NA	NA	(Grioni et al., 2019)
	NA[a]	NA	NA	NA	B-ALL	NA	NA	(Chen et al., 2021)
**PCM1 (8p22)**	t(8;9)(p22;p24)	exon 9	exon 36	M/50	B-ALL	NA	No	(Reiter et al., 2005)
	NA[a]	NA	NA	M/NA	B-ALL	NA	NA	(Reshmi et al., 2017)
	t(8;9)(p21;p24)	exon 11	exon 35	F/15	T-ALL	NA	Yes/No	(Kaplan et al., 2021)
**PPFIBP1 (12p11.2)**	NA[a]	exon 19	exon 12	M/21-39	B-ALL	Yes	NA	(Roberts et al., 2014), (Roberts et al., 2017)
**RFX3 (9p24.2)**	t(9;9)(p24.1;p24.2)	NA	NA	F/NA	B-ALL	NA	NA	(Reshmi et al., 2017)
**RNPC3 (1p21.1)**	t(1;9)(p13;p22)	exon 13	exon 13	F/29	B-ALL	Yes	Yes/Yes	(Chen et al., 2019)
	NA[a]	NA	NA	NA	B-ALL	NA	NA	(Chen et al., 2021)
**ROCK1 (18q11.1)**	NA[a]	NA	NA	NA	B-ALL	NA	NA	(Chen et al., 2021)
**SMU1 (9p21.1)**	NA[a]	exon 13	exon 6	F/60-86	B-ALL	Yes	NA	(Roberts et al., 2017)
**SNX29 (16p13.13-p13.12)**	NA[a]	NA	NA	NA	B-ALL	NA	NA	(Tasian et al., 2017)
	NA[a]	NA	NA	NA	B-ALL	NA	NA	(Chen et al., 2021)
**SPAG9 (17q21.3)**	t(9;17)(p24;q21)	exon 19	exon 25	M/14	B-ALL	Yes	Yes/Yes	(Kawamura et al., 2015)
**SSBP2 (5q14.1)**	t(5;9)(p14.1;p24.1)	exon 11	exon 4	M/39	B-ALL	NA	Yes/Yes	(Poitras et al., 2008)
	NA[a]	exon 18	exon 8	F/21-39	B-ALL	Yes	NA	(Roberts et al., 2014), (Roberts et al., 2017)

	t(5;9)(q12;p1?3)	exon 18	exon 10	M/14	B-ALL	Yes	Yes/NA	(Roberts et al., 2014)
	NA[a]	exon 17	exon 8	F/40-59	B-ALL	Yes	NA	(Roberts et al., 2017)
	NA[a]	NA	NA	M/NA	B-ALL	NA	NA	(Reshmi et al., 2017)
	NA[a]	NA	NA	F/NA	B-ALL	NA	NA	(Reshmi et al., 2017)
**STRBP**	inv(9)(p24;q33) or t(9;9)(p24;q33)	exon 19	exon 18	F/21	B-ALL	No	yes/yes	(Zhang et al., 2020)
**STRN3 (14q12)**	NA[a]	exon 17	exon 9	F/12.2	B-ALL	Yes	NA	(Roberts et al., 2012)
	NA[a]	exon 17	exon 9	F/10-15	B-ALL	Yes	NA	(Roberts et al., 2014)
**TBL1XR1 (3q26.32)**	t(3;9)(q26;p24)	exon 14	exon 14	M/5	T-ALL	NA	Yes/No	(Huang et al., 2020)
**TERF2 (16q22.1)**	NA[a]	exon 19	exon 8	F/16-20	B-ALL	Yes	NA	(Roberts et al., 2014)
	NA[a]	exon 19	exon 10	M/NA	B-ALL	No	Yes/Yes	(Boer et al., 2017)
	NA[a]	exon 19	exon 10	NA/11	B-ALL	NA	Yes/Yes	(Steeghs et al., 2017)
**TPM3 (1q21.3)**	NA[a]	exon 17	exon 7	M/23	T-ALL	NA	Yes/Yes	(Kalender Atak et al., 2013)
**TPR (1q31.1)**	NA[a]	exon 17	exon 39	M/21-39	B-ALL	Yes	NA	(Roberts et al., 2014)
**USP25 (21q21.1)**	NA[a]	NA	NA	M/NA	B-ALL	NA	NA	(Reshmi et al., 2017)
**ZBTB20 (3q13.3)**	t(3;9)(q13;p24)	exon 19	exon 4	F/33	B-ALL	Yes	NA	(Peterson et al., 2019)
	t(3;9)(q13;p24)	exon 19	exon 4	F/33	B-ALL	Yes	Yes/No	(Lee et al., 2020)
**ZBTB46/ZNF340 (20q13.33)**	NA	NA	NA	NA	B-ALL	NA	NA	(Tasian et al., 2017)
	NA[a]	exon 19	exon 4	F/40-59	B-ALL	Yes	NA	(Roberts et al., 2017)
**ZEB2 (2q22.3)**	t(2;9)(p24.1;q22.3)	NA	NA	M/NA	B-ALL	NA	HR4	(Grioni et al., 2019)
**ZNF274 (19q13.43)**	NA[a]	NA	NA	F/NA	B-ALL	NA	NA	(Reshmi et al., 2017)

CR, complete remission; NA, not available; B-ALL, B-cell acute lymphoblastic leukaemia; T-ALL, T-cell acute lymphoblastic leukaemia.

[a]Fusion gene not identified by cytogenetic analysis: karyotype may be complex, or the rearrangement may be cytogenetically cryptic.
[b]Identified by multiplex-nested PCR, reverse primer bound within *JAK2* exon 16
[c]Reciprocal *JAK2-PAX5* fusion where *PAX5* comprises the C-terminus of the fusion gene.

Supplementary Table II. Fusion partner genes of known *JAK2* fusions and the normal function of the resultant proteins. Gene summary information was acquired from GeneCards – the human gene database (Stelzer et al., 2016). Proteins known to form dimers are indicated along with potential domains that when present in a JAK2 fusion oligomerization are shown; CC, colied-coil; HLH, helix-loop-helix; Homeo, homeodomain; LisH, lis homology domain; RFXO, RFX oligomerization domain; DZF, zinc-finger dimerisation domain; dsRBD, double-stranded RNA-binding domain; TRFH, TERF homology domain; BTB, tkk and bab domain; scan, scan motif.

Fusion Partner	Gene Name	Expression in B-cells	WT Dimer	Function of Protein	Potential Oligo. Domain
**ATF7IP**	Activating transcription factor 7 interacting protein	Low		transcription factor	Unknown
**BCR**	Breakpoint cluster region	low	Yes	GTPase-activating protein	CC
**EBF1**	Early B-cell factor 1	high	Yes	transcription factor	HLH
**ERC1**	ELKS/RAB6-Interacting/CAST Family Member 1	low	Yes	Scaffold protein that promotes cell migration	CC
**ETV6**	ETS translocation variant 6	high	Yes	transcription factor	HLH
**GOLGA4/5**	Golgin subfamily A, 4/5	low	Yes	vesicle tethering and docking at the Golgi	CC
**HMBOX1**	Homeobox containing 1	none		enables binding of telomerase complex to DNA	Homeo
**MPRIP**	Myosin phosphatase Rho interacting protein	low		targets myosin phosphatase to the actin cytoskeleton	CC
**NPHP3**	Nephrocystin 3	none		Associated with ciliary development	CC
**OFD1**	Oral facial digital syndrome 1	low		component of centrioles	LisH
**PAX5**	Paired box 5	high		transcription factor	HLH
**PCM1**	Pericentriolar material 1	low		component of centrioles	CC

**PPFIBP1**	PTPRF interacting protein 1	none		LAR protein tyrosine phosphatase	CC
**RFX3**	Regulatory factor X3	low	Yes	transcription factor	RFXO
**RNPC3**	RNA binding region containing protein 3	low		component of the U12-type spliceosome	Unknown
**ROCK1**	Rho associated coiled-coil containing protein kinase 1	high	Yes	Regulates actin cytoskeleton and cell polarity	CC
**SMU1**	SMU1 DNA replication regulator and spliceosomal factor	high	Yes	component of the spliceosome	LisH
**SNX29**	Sorting nexin 29	high	Yes	associated with microtubule motor activity	CC
**SPAG9**	Sperm associated antigen 9	low		scaffold protein	CC
**SSBP2**	Single-stranded DNA binding protein 2	low	Yes	associated with the DNA damage response and genome stability	LisH
**STRBP**	Spermatid perinuclear RNA binding protein	high	Yes	Associated with spermatogenesis and sperm function	DZF or dsRBD
**STRN3**	Striatin 3	high	Yes	associated with calmodulin binding	CC
**TBL1XR1**	TBL1X receptor 1	high	Yes	Co-activator of the androgen receptor	LisH
**TERF2**	Telomeric repeat binding factor 2	high	Yes	component of the telomere nucleoprotein complex	TRFH
**TPM3**	Tropomyosin 3	high	Yes	regulates actin filament stability	CC
**TPR**	Translocated promoter region	high	Yes	component of intranuclear filaments of nuclear pore complexes (NPCs)	CC
**USP25**	Ubiquitin specific peptidase 25	low	Yes	Degrades ubiquitin-tagged proteins	CC

**ZBTB20**	Zinc finger and BTB domain containing protein 20	none	Yes	transcription factor	BTB
**ZBTB46**	Zinc finger and BTB domain containing protein 46	none	Yes	transcription factor	BTB
**ZEB2**	Zinc finger E-box binding homeobox 2	low	Yes	transcriptional repressor	Homeo
**ZNF274**	Zinc finger protein 274	none	Yes	transcriptional repressor	scan

Supplementary References

Boer, J.M., Steeghs, E.M.P., Marchante, J.R.M., Boeree, A., Beaudoin, J.J., Beverloo, H.B., Kuiper, R.P., Escherich, G., Velden, V.H.J.V.D., Schoot, C.E.V.D., Kruseman, H.a.D.G.-., Pieters, R., and Boer, M.L.D. (2017). Tyrosine kinase fusion genes in pediatric BCR-ABL1-like acute lymphoblastic leukemia. *Oncotarget* 8**,** 4618-4628.

Chen, X., Wang, F., Zhang, Y., Ma, X., Cao, P., Yuan, L., Wang, L., Chen, J., Zhou, X., Wu, Q., Liu, M., Jin, D., and Liu, H. (2021). Fusion gene map of acute leukemia revealed by transcriptome sequencing of a consecutive cohort of 1000 cases in a single center. *Blood Cancer J* 11**,** 112.

Chen, X., Wang, F., Zhang, Y., Ma, X., Liu, M., Cao, P., Zhou, L., Wang, L., Zhang, X., Wang, T., and Liu, H. (2019). Identification of *RNPC3* as a novel *JAK2* fusion partner gene in B-acute lymphoblastic leukemia refractory to combination therapy including ruxolitinib. *Molecular Genetics & Genomic Medicine* 8**,** e1110.

Coyaud, E., Struski, S., Prade, N., Familiades, J., Eichner, R., Quelen, C., Bousquet, M., Mugneret, F., Talmant, P., Pages, M.-P., Lefebvre, C., Penther, D., Lippert, E., Nadal, N., Taviaux, S., Poppe, B., Luquet, I., Baranger, L., Eclache, V., Radford, I., Barin, C., Mozziconacci, M.-J., Lafage-Pochitaloff, M., Antoine-Poirel, H., Charrin, C., Perot, C., Terre, C., Brousset, P., Dastugue, N., and Broccardo, C. (2010). Wide diversity of PAX5 alterations in B-ALL: a Groupe Francophone de Cytogénétique Hématologique study. *Blood* 115**,** 3089-3097.

Ding, Y.Y., Stern, J.W., Jubelirer, T.F., Wertheim, G.B., Li, F., Chang, F., Gu, Z., Mullighan, C.G., Li, Y., Harvey, R.C., Chen, I.M., Willman, C.L., Hunger, S.P., Li, M.M., and Tasian, S.K. (2018). Clinical efficacy of ruxolitinib and chemotherapy in a child with Philadelphia chromosome-like acute lymphoblastic leukemia with GOLGA5-JAK2 fusion and induction failure. *Haematologica* 103**,** e427-e431.

Downes, C.E.J., Rehn, J., Heatley, S.L., Yeung, D., Mcclure, B.J., and White, D.L. (2021). Identification of a novel *GOLGA4-JAK2* fusion gene in B-cell acute lymphoblastic leukaemia. *Br J Haematol***,** Advance online publication.

Duployez, N., Nibourel, O., Ducourneau, B., Grardel, N., Boyer, T., Bories, C., Darre, S., Coiteux, V., Berthon, C., Preudhomme, C., and Roche-Lestienne, C. (2016). Acquisition of genomic events leading to lymphoblastic transformation in a rare case of myeloproliferative neoplasm with *BCR-JAK2* fusion transcript. *European Journal of Haematology* 97**,** 399-402.

Grioni, A., Fazio, G., Rigamonti, S., Bystry, V., Daniele, G., Dostalova, Z., Quadri, M., Saitta, C., Silvestri, D., Songia, S., Storlazzi, C.T., Biondi, A., Darzentas, N., and Cazzaniga, G. (2019). A simple RNA target capture NGS strategy for fusion genes assessment in the diagnostics of pediatric B-cell acute lymphoblastic leukemia. *HemaSphere* 3**,** e250.

Huang, X., Celiker, M., Guarini, L., Patel, S., and Chen, N.N. (2020). TBL1XR1-JAK2: a novel fusion in a pediatric T cell acute lymphoblastic leukemia patient with increased absolute eosinophil count. *Journal of Hematopathology* 13**,** 259-263.

Imamura, T., Kiyokawa, N., Kato, M., Imai, C., Okamoto, Y., Yano, M., Ohki, K., Yamashita, Y., Kodama, Y., Saito, A., Mori, M., Ishimaru, S., Deguchi, T., Hashii, Y., Shimomura, Y., Hori, T., Kato, K., Goto, H., Ogawa, C., Koh, K., Taki, T., Manabe, A., Sato, A., Kikuta, A., Adachi, S., Horibe, K., Ohara, A., Watanabe, A., Kawano, Y., Ishii, E., and Shimada, H. (2016). Characterization of pediatric Philadelphia-negative B-cell precursor acute lymphoblastic leukemia with kinase fusions in Japan. *Blood Cancer Journal* 6**,** e419.

Kalender Atak, Z., Gianfelici, V., Hulselmans, G., De Keersmaecker, K., Devasia, A.G., Geerdens, E., Mentens, N., Chiaretti, S., Durinck, K., Uyttebroeck, A., Vandenberghe, P., Wlodarska, I., Cloos, J., Foà, R., Speleman, F., Cools, J., and Aerts, S. (2013). Comprehensive analysis of transcriptome variation uncovers known and novel driver events in T-cell acute lymphoblastic leukemia. *PLoS Genetics* 9**,** e1003997

Kaplan, H.G., Bifulco, C.B., Jin, R., Scanlan, J.M., and Corwin, D. (2021). Treatment of PCM1-JAK2 fusion tyrosine kinase gene-related acute lymphoblastic leukemia with stem cell transplantation. *Future Rare Diseases* 1**,** FRD10.

Kawamura, M., Taki, T., Kaku, H., Ohki, K., and Hayashi, Y. (2015). Identification of *SPAG9* as a novel *JAK2* fusion partner gene in pediatric acute lymphoblastic leukemia with t(9;17)(p24;q21). *Genes, Chromosomes & Cancer* 54**,** 401-408.

Lacronique, V., Boureux, A., Valle, V., Poirel, H., Quang, C., Mauchauffé, M., Berthou, C., Lessard, M., Berger, R., Ghysdael, J., and Bernard, O. (1997). A TEL-JAK2 fusion protein with constitutive kinase activity in human leukemia. *Science* 278**,** 1309-1312.

Lee, W.Y., Pfau, R.B., Choi, S.M., Yang, J., Xiao, H., Putnam, E.M., Ryan, R.J., Bixby, D.L., and Shao, L. (2020). The diagnostic challenges and clinical course of a myeloid/lymphoid neoplasm with eosinophilia and *ZBTB20-JAK2* gene fusion presenting as B-lymphoblastic leukemia. *Cold Spring Harbor Molecular Case Studies* 6**,** a004937.

Nebral, K., Denk, D., Attarbaschi, A., Konig, M., Mann, G., Haas, O.A., and Strehl, S. (2009). Incidence and diversity of *PAX5* fusion genes in childhood acute lymphoblastic leukemia. *Leukemia* 23**,** 134-143.

Peeters, P., Raynaud, S.D., Cools, J., Wlodarska, I., Grosgeorge, J., Philip, P., Monpoux, F., Rompaey, L.V., Baens, M., Berghe, H.V.D., and Marynen, P. (1997). Fusion of TEL, the ETS-variant gene 6 (ETV6), to the receptor-associated kinase JAK2 as a result of t(9;12) in a lymphoid and t(9;15;12) in a myeloid leukemia. *Blood* 90**,** 2535-2540.

Peterson, J.F., Blackburn, P.R., Webley, M.R., Pearce, K.E., Williamson, C.M., Vasmatzis, G., Smadbeck, J.B., Bieliauskas, S.L., Reichard, K.K., Ketterling, R.P., Baughn, L.B., and Greipp, P.T. (2019). Identification of a novel *ZBTB20-JAK2* fusion by mate-pair sequencing in a young adult with B-lymphoblastic leukemia/lymphoma. *Mayo Clinic Proceedings* 94**,** 1381-1384.

Poitras, J.L., Dal Cin, P., Aster, J.C., Deangelo, D.J., and Morton, C.C. (2008). Novel *SSBP2-JAK2* fusion gene resulting from a t(5;9)(q14.1;p24.1) in pre-B acute lymphocytic leukemia. *Genes Chromosomes Cancer* 47**,** 884-889.

Reiter, A., Walz, C., Watmore, A., Schoch, C., Blau, I., Schlegelberger, B., Berger, U., Telford, N., Aruliah, S., Yin, J.A., Vanstraelen, D., Barker, H.F., Taylor, P.C., O'driscoll, A., Benedetti, F., Rudolph, C., Kolb, H.-J., Hochhaus, A., Hehlmann, R., Chase, A., and Cross, N.C.P. (2005). The t(8;9)(p22;p24) is a recurrent abnormality in chronic and acute leukemia that fuses PCM1 to JAK2. *Cancer Research* 65**,** 2662-2667.

Reshmi, S.C., Harvey, R.C., Roberts, K.G., Stonerock, E., Smith, A., Jenkins, H., Chen, I.M., Valentine, M., Liu, Y., Li, Y., Shao, Y., Easton, J., Payne-Turner, D., Gu, Z., Tran, T.H., Nguyen, J.V., Devidas, M., Dai, Y., Heerema, N.A., Carroll, A.J., 3rd, Raetz, E.A., Borowitz, M.J., Wood, B.L., Angiolillo, A.L., Burke, M.J., Salzer, W.L., Zweidler-Mckay, P.A., Rabin, K.R., Carroll, W.L., Zhang, J., Loh, M.L., Mullighan, C.G., Willman, C.L., Gastier-Foster, J.M., and Hunger, S.P. (2017). Targetable kinase gene fusions in high-risk B-ALL: a study from the Children's Oncology Group. *Blood* 129**,** 3352-3361.

Roberts, K.G., Gu, Z., Payne-Turner, D., Mccastlain, K., Harvey, R.C., Chen, I.M., Pei, D., Iacobucci, I., Valentine, M., Pounds, S.B., Shi, L., Li, Y., Zhang, J., Cheng, C., Rambaldi, A., Tosi, M., Spinelli, O., Radich, J.P., Minden, M.D., Rowe, J.M., Luger, S., Litzow, M.R., Tallman, M.S., Wiernik, P.H., Bhatia, R., Aldoss, I., Kohlschmidt, J., Mrozek, K., Marcucci, G., Bloomfield, C.D., Stock, W., Kornblau, S., Kantarjian, H.M., Konopleva, M., Paietta, E., Willman, C.L., and Mullighan, C.G. (2017). High frequency and poor outcome of Philadelphia chromosome-like acute lymphoblastic leukemia in adults. *Journal of Clinical Oncology* 35**,** 394-401.

Roberts, K.G., Li, Y., Payne-Turner, D., Harvey, R.C., Yang, Y.L., Pei, D., Mccastlain, K., Ding, L., Lu, C., Song, G., Ma, J., Becksfort, J., Rusch, M., Chen, S.C., Easton, J., Cheng, J., Boggs, K., Santiago-Morales, N., Iacobucci, I., Fulton, R.S., Wen, J., Valentine, M., Cheng, C., Paugh, S.W., Devidas, M., Chen, I.M., Reshmi, S., Smith, A., Hedlund, E., Gupta, P.,

Nagahawatte, P., Wu, G., Chen, X., Yergeau, D., Vadodaria, B., Mulder, H., Winick, N.J., Larsen, E.C., Carroll, W.L., Heerema, N.A., Carroll, A.J., Grayson, G., Tasian, S.K., Moore, A.S., Keller, F., Frei-Jones, M., Whitlock, J.A., Raetz, E.A., White, D.L., Hughes, T.P., Guidry Auvil, J.M., Smith, M.A., Marcucci, G., Bloomfield, C.D., Mrozek, K., Kohlschmidt, J., Stock, W., Kornblau, S.M., Konopleva, M., Paietta, E., Pui, C.H., Jeha, S., Relling, M.V., Evans, W.E., Gerhard, D.S., Gastier-Foster, J.M., Mardis, E., Wilson, R.K., Loh, M.L., Downing, J.R., Hunger, S.P., Willman, C.L., Zhang, J., and Mullighan, C.G. (2014). Targetable kinase-activating lesions in Ph-like acute lymphoblastic leukemia. *New England Journal of Medicine* 371**,** 1005-1015.

Roberts, K.G., Morin, R.D., Zhang, J., Hirst, M., Zhao, Y., Su, X., Chen, S.-C., Payne-Turner, D., Churchman, M.L., Harvey, Richard c., Chen, X., Kasap, C., Yan, C., Becksfort, J., Finney, Richard p., Teachey, David t., Maude, Shannon l., Tse, K., Moore, R., Jones, S., Mungall, K., Birol, I., Edmonson, Michael n., Hu, Y., Buetow, Kenneth e., Chen, I.M., Carroll, William l., Wei, L., Ma, J., Kleppe, M., Levine, Ross l., Garcia-Manero, G., Larsen, E., Shah, Neil p., Devidas, M., Reaman, G., Smith, M., Paugh, Steven w., Evans, William e., Grupp, Stephan a., Jeha, S., Pui, C.-H., Gerhard, Daniela s., Downing, James r., Willman, Cheryl l., Loh, M., Hunger, Stephen p., Marra, Marco a., and Mullighan, Charles g. (2012). Genetic alterations activating kinase and cytokine receptor signaling in high-risk acute lymphoblastic leukemia. *Cancer Cell* 22**,** 153-166.

Roberts, K.G., Reshmi, S.C., Harvey, R.C., Chen, I.-M., Patel, K., Stonerock, E., Jenkins, H., Dai, Y., Valentine, M., Gu, Z., Zhao, Y., Zhang, J., Payne-Turner, D., Devidas, M., Heerema, N.A., Carroll, A.J., Raetz, E.A., Borowitz, M.J., Wood, B.L., Jr, L.a.M., Maloney, K.W., Carroll, W.L., Loh, M.L., Willman, C.L., Gastier-Foster, J.M., Mullighan, C.G., and Hunger, S.P. (2018). Genomic and outcome analyses of Ph-like ALL in NCI standard-risk patients: a report from the Children's Oncology Group. *Blood* 132**,** 815-824.

Steeghs, E.M.P., Jerchel, I.S., Goffau-Nobel, W.D., Hoogkamer, A.Q., Boer, J.M., Boeree, A.L., Ven, C.V.D., Koudijs, M.J., Besselink, N.J.M., Groot-Kruseman, H.a.D., Zwaan, C.M., Horstmann, M.A., Pieters, R., and Boer, M.L.D. (2017). JAK2 aberrations in childhood B-cell precursor acute lymphoblastic leukemia. *Oncotarget* 8**,** 89923-89938.

Stelzer, G., Rosen, R., Plaschkes, I., Zimmerman, S., Twik, M., Fishilevich, S., Stein, T., Nudel, R., Lieder, I., Mazor, Y., Kaplan, S., Dahary, D., Warshawsky, D., Guan-Golan, Y., Kohn, A., Rappaport, N., Safran, M., and Lancet, D. (2016). The GeneCards Suite: From Gene Data Mining to Disease Genome Sequence Analysis. *Current Protocols in Bioinformatics* 54**,** 1.30.31-31.30.33.

Tasian, S.K., Loh, M.L., and Hunger, S.P. (2017). Philadelphia chromosome–like acute lymphoblastic leukemia. *Blood* 130**,** 2064-2072.

Tirado, C.A., Chen, W., Huang, L.J., Laborde, C., Hiemenz, M.C., Valdez, F., Ho, K., Winick, N., Lou, Z., and Koduru, P. (2010). Novel JAK2 rearrangement resulting from a t(9;22)(p24;q11.2) in B-acute lymphoblastic leukemia. *Leukemia Research* 34**,** 1674-1676.

Tirado, C.A., Shabsovich, D., Denicola, M., Rao, D., Yang, L., Garcia, R., and Rao, N. (2013). A case of pediatric B-Lymphoblastic leukemia presenting with a t(9;12)(p24;q11.2) involving *JAK2* and concomitant *MLL* rearrangement with apparent insertion at 6q27. *Biomarker Research* 1.

Yano, M., Imamura, T., Asai, D., Kiyokawa, N., Nakabayashi, K., Matsumoto, K., Deguchi, T., Hashii, Y., Honda, Y., Hasegawa, D., Sasahara, Y., Ishii, M., Kosaka, Y., Kato, K., M.Shima, Hori, H., Yumura-Yagi, K., Hara, J., Oda, M., Horibe, K., Ichikawa, H., and Sato, A. (2015). Identification of novel kinase fusion transcripts in paediatric B cell precursor acute lymphoblastic leukaemia with IKZF1 deletion. *British Journal of Haematology* 151**,** 813-817.

Zhang, X.Y., Dai, H.P., Li, Z., Yin, J., Lang, X.P., Yang, C.X., Xiao, S., Zhu, M.Q., Liu, D.D., Liu, H., Shen, H.J., Wu, D.P., and Tang, X.W. (2020). Identification of STRBP as a Novel JAK2

Fusion Partner Gene in a Young Adult With Philadelphia Chromosome-Like B-Lymphoblastic Leukemia. *Front Oncol* 10, 611467.
Zhou, M.H., Gao, L., Jing, Y., Xu, Y.Y., Ding, Y., Wang, N., Wang, W., Li, M.Y., Han, X.P., Sun, J.Z., Wang, L.L., and Yu, L. (2012). Detection of *ETV6* gene rearrangements in adult acute lymphoblastic leukemia. *Annals of Hematology* 91, 1235-1243.
